# 4D-Printed Elution-Peak-Guided Dual-Responsive Monolithic Packing for the Solid-Phase Extraction of Metal Ions

**DOI:** 10.1021/acs.analchem.3c04961

**Published:** 2024-02-21

**Authors:** Wen-Hsiu Tsai, Cheng-Kuan Su

**Affiliations:** Department of Chemistry, National Chung Hsing University, Taichung 402, Taiwan, R.O.C

## Abstract

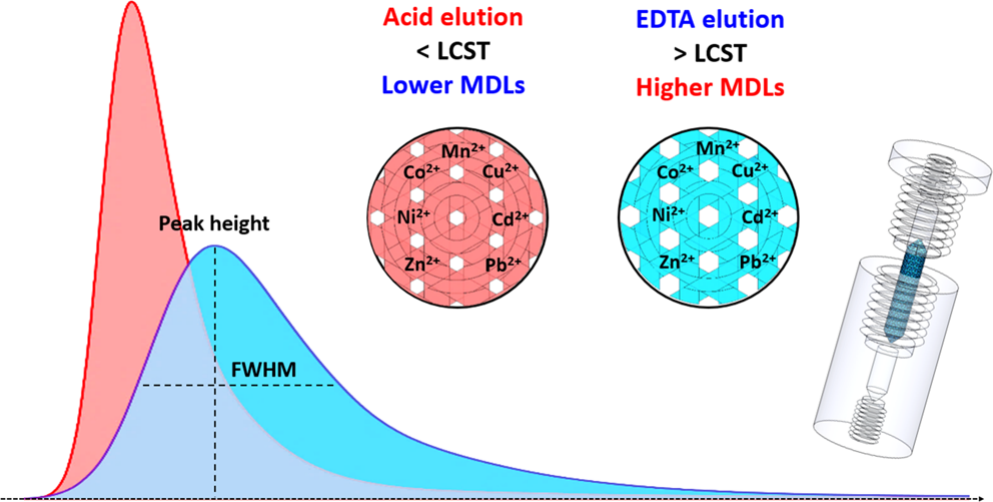

Four-dimensional printing (4DP) technologies are revolutionizing the fabrication of stimuli-responsive devices. To advance the analytical performance of conventional solid-phase extraction (SPE) devices using 4DP technology, in this study, we employed *N*-isopropylacrylamide (NIPAM)-incorporated photocurable resins and digital light processing three-dimensional printing to fabricate an SPE column with a [H^+^]/temperature dual-responsive monolithic packing stacked as interlacing cuboids to extract Mn, Co, Ni, Cu, Zn, Cd, and Pb ions. When these metal ions were eluted using 0.5% HNO_3_ solution as the eluent at a temperature below the lower critical solution temperature of polyNIPAM, the monolithic packing swelled owing to its hydrophilic/hydrophobic transition and electrostatic repulsion among the protonated units of polyNIPAM. These effects resulted in smaller interstitial volumes among these interlacing cuboids and improvements in the elution peak profiles of the metal ions, which, in turn, demonstrated the reduced method detection limits (MDLs; range, 0.2–7.2 ng L^–1^) during analysis using inductively coupled plasma mass spectrometry. We studied the effects of optimizing the elution peak profiles of the metal ions on the analytical performance of this method and validated its reliability and applicability by analyzing the metal ions in reference materials (CASS-4, SLRS-5, 1643f, and Seronorm Trace Elements Urine L-2) and performing spike analyses of seawater, groundwater, river water, and human urine samples. Our results suggest that this 4D-printed elution-peak-guided dual-responsive monolithic packing enables lower MDLs when packed in an SPE column to facilitate the analyses of the metal ions in complex real samples.

## Introduction

Trace metals serve as indispensable indicators of essential nutrients and potential toxins and are significant in environmental and biomedical studies.^1−3^ To determine these metals in complex real samples (*e.g*., seawater, urine, and blood) is often difficult using atomic spectrometric techniques [*e.g*., inductively coupled plasma mass spectrometry (ICP-MS)] because unpredictable biases and spectral and nonspectral interferences could contribute to the poor reliability of the analytical results.^4−6^ Thus, sample preparation, which aims to eliminate sample matrices and/or enrich target analytes, is necessary and critically determines the applicability of modern analytical techniques, especially for reliable quantitative analysis.^7,8^ Solid-phase extraction (SPE), which employs sorbent-filled columns or cartridges to selectively extract target analytes from complicated sample matrices, is routinely used as an efficient sample-pretreatment scheme to improve the reliability of trace-metal analysis when using atomic spectrometric methods.^9−11^

Enhancing the extraction efficiencies of the column packing for target analytes is considered a preliminary strategy to improve the analytical performance of conventional SPE schemes.^12,13^ Given their large specific surface areas and excellent extraction capacities, many porous materials have been packed in SPE devices to enhance the extraction efficiencies of the column or cartridge for target analytes and even approach near-complete extraction.^14−16^ However, complex porous structures could frequently lead to undesirable analytical performance, such as elution-peak tailing and significant carry-over effects.^15,17,18^ In addition, if the blank level is significantly higher than the baseline noise, the use of porous sorbents to extract metal ions could enhance not only the signal intensities of the analytes but also those of the blank, leading to no benefit or improvement in the detection capabilities of the analytical method after the laborious process of enhancing the extraction efficiencies of the packing for target analytes.

In theory, the method detection limit (MDL), which is defined as 3 times the standard deviation of the baseline noise determined from seven blank measurements,^19,20^ can be improved by increasing the sensitivity of the packing toward the target analytes and/or reducing the blank level to achieve lower variations from baseline noise, thus enhancing the detection capability of an analytical method.^20−22^ If enhancement of the sensitivity of the packing toward target analytes is no longer technically affordable, improving the elution peak profile of the target analytes, which is represented by the full width at half-maximum (fwhm),^23,24^ could provide more discrimination between the analyte and blank signals under the same elution peak area. This strategy could improve the signal-to-noise ratio and be potentially useful for reducing MDLs, especially when analyzing metal ions with negligible blank levels. Owing to limitations in the availability of tools and techniques to develop analytical devices with desirable geometric features and functions, few studies have focused on optimizing the elution peak profiles of the target analytes.^25,26^ Challenges in devising SPE sorbents and devices to optimize the elution peak profiles of metal ions and improve the analytical performance (*e.g*., signal-to-noise ratios, MDLs) of SPE-based sample-pretreatment schemes for trace-metal analysis remain.

Owing to their inherent properties, stimuli-responsive materials have been utilized to improve the extraction efficiencies of SPE devices for target analytes and develop new separation strategies.^27−31^ For example, Nagase et al. examined the effect of the pore diameter (7, 12, and 30 nm) of silica beads on the elution behavior of analytes on a chromatographic column packed with a temperature-responsive copolymer hydrogel and observed temperature-dependent elution behaviors (retention times) owing to the temperature-responsive properties of the hydrogel and analyte diffusion into the bead pores.^30^ An et al. realized light-driven polarity switching in a capillary gas chromatographic column based on the reversible *trans*–*cis* isomerization of the stationary phase under light irradiation and showed that this photosensitive column exhibited good polarity photoreversibility for high separation efficiency.^31^ However, stimuli-responsive materials have never been used to improve the (elution) peak profiles of target analytes.

Three-dimensional printing (3DP) technologies are highly applicable to the customization of diverse analytical devices featuring special geometric characteristics or functionality.^32−37^ The coupling of 3D-printed SPE devices with atomic spectrometric techniques has been demonstrated to allow improvements in adaptability, diversity, and analytical performance for trace-metal analysis.^38−48^ Moreover, emerging four-dimensional printing (4DP) technologies based on the 3DP of stimuli-responsive materials are capable of effectively fabricating stimuli-responsive analytical devices possessing time-dependent shape programming and/or shape-memory properties.^49−53^ At present, studies utilizing stimuli-responsive materials to improve the analytical performance of conventional SPE schemes are limited. It is necessary to explore the capability of 4DP technologies for advancing SPE-based analytical schemes to develop future sample-preparation techniques.

The aim of this study is to employ 4DP technologies to fabricate stimuli-responsive SPE sorbents that could improve the elution peak profiles of metal ions and achieve lower MDLs. Herein, we used *N*-isopropylacrylamide (NIPAM)-incorporated photocurable resins and digital light processing (DLP) 3DP to fabricate an SPE column featuring a [H^+^]/temperature dual-responsive monolithic packing to extract Mn, Co, Ni, Cu, Zn, Cd, and Pb ions prior to ICP-MS determination. When the metal ions extracted by the fabricated monolithic packing were eluted with 0.5% HNO_3_ solution as the eluent at a temperature below the lower critical solution temperature (LCST) of polyNIPAM, the monolithic packing swelled owing to its temperature-controlled hydrophilic/hydrophobic transition^54,55^ and electrostatic repulsion among the protonated units (NH_2_^+^; when pH < 3.0)^56−60^ of polyNIPAM. These effects resulted in smaller interstitial volumes among the interlacing cuboids and improvements in the elution peak profiles of the metal ions. Unlike conventional SPE schemes that are usually improved by increasing their extraction efficiency and optimized based on the signal intensities of the target analytes (*i.e*., their sensitivity), we focused on the use of stimuli-responsive materials to optimize the elution peak profiles of the metal ions to achieve lower MDLs. Following the optimization of the design and fabrication of the SPE column with the dual-responsive monolithic packing, extraction conditions, and automatic analytical system, we validated the reliability and applicability of our method by analyzing the metal ions in reference materials (CASS-4, SLRS-5, 1643f, and Seronorm Trace Elements Urine L-2) and performing spike analyses of collected samples (seawater, groundwater, river water, and human urine) using ICP-MS. To the best of our knowledge, this 4D-printed elution-peak-guided dual-responsive monolithic packing is the first to provide an optimized SPE scheme that improves the elution peak profiles but not the elution peak areas of metal ions, thereby highlighting the promising capability of 4DP technologies in advancing the analytical performance of conventional analytical devices.

## Experimental Section

### Chemicals

NIPAM (415324), *tert*-butyl acrylate (tBA; 327182), 1,6-hexanediol diacrylate (HDDA; 246816), diphenyl(2,4,6-trimethylbenzoyl)phosphine oxide (TPO; 415952), and disodium hydrogen phosphate (Na_2_HPO_4_; 71629, TraceSELECT grade) were purchased from Sigma–Aldrich. Nitric acid (HNO_3_; 6901, ultrapure grade) was purchased from J.T. Baker. Sodium hydroxide (106466, Suprapur grade) was purchased from Merck for pH adjustment. All chemical solutions were prepared with water purified using a Milli-Q IQ 7000 water purification system (Merck Millipore). Working solutions were prepared *via* the serial dilution of multielement calibration standards of Mn(II), Co(II), Ni(II), Cu(II), Zn(II), Cd(II), and Pb(II) (10 mg L^–1^; N9300233, PerkinElmer) with 10 mM phosphate buffer. Photocurable resins of the dual-responsive polymers were prepared by mixing NIPAM, tBA, HDDA, and TPO (25:56:18:1). TPO was used as the photoinitiator, NIPAM was used for its stimuli-responsive properties,^54−60^ and tBA and HDDA were used for their shape-memory property.^46,61^

### SPE Column with the 4D-Printed Dual-Responsive Monolithic Packing

**Figure 1:**
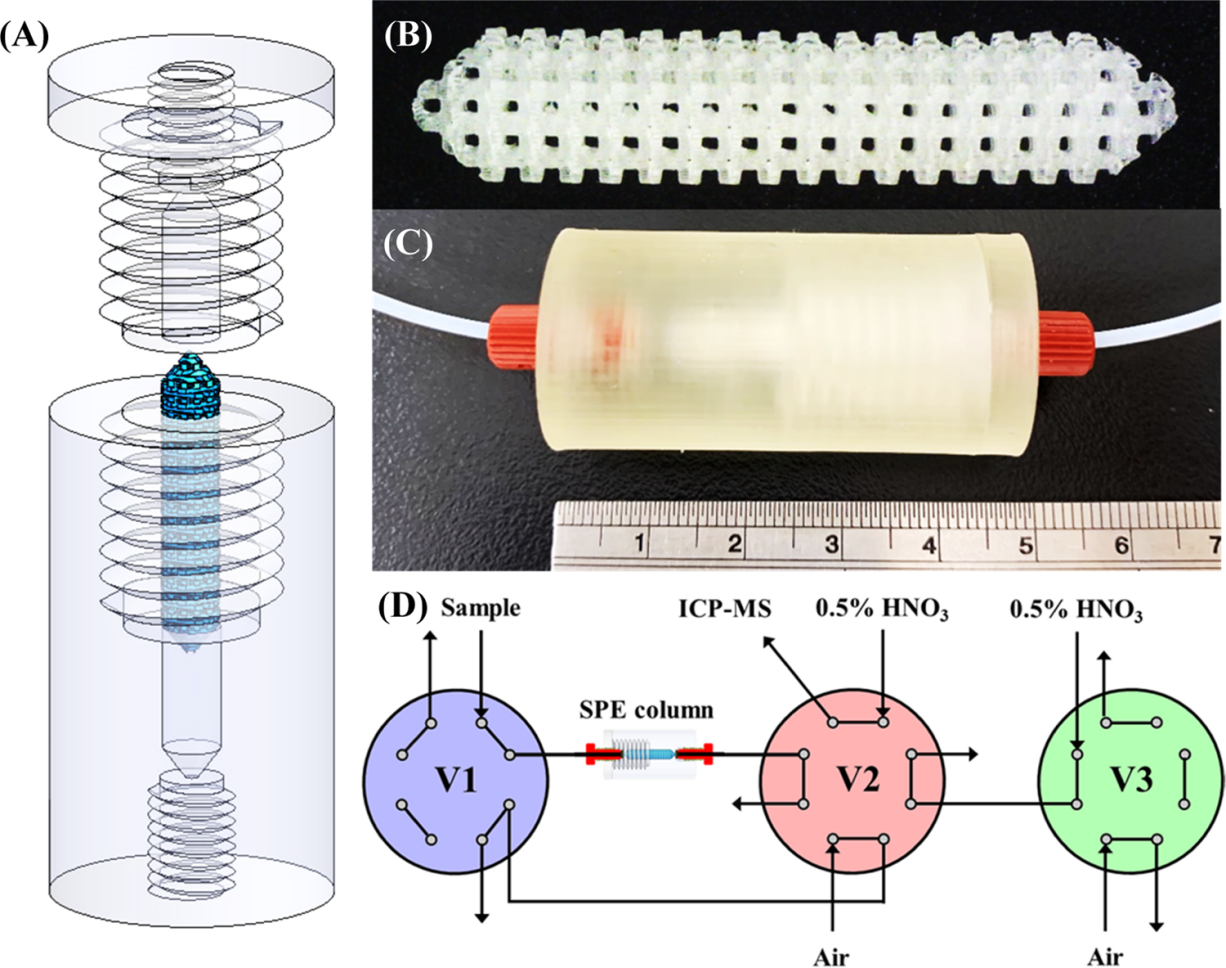
(A) CAD drawing of the SPE column containing the column body (gray; fabricated using BV-007A photocurable resins) and monolithic packing (blue; fabricated using NIPAM-incorporated photocurable resins). (B) Photograph of the 4D-printed elution-peak-guided dual-responsive monolithic packing. (C) Photograph of the fabricated SPE column (including the monolithic packing) fitted with two flat-bottom male connectors. (D) Schematic representation of the automatic analytical system featuring the SPE column with the 4D-printed dual-responsive monolithic packing. V1, V2, and V3: two-position, eight-port rotary valves; arrows: outflow of liquid waste.

The 3D object of the SPE column (Figure 1A) was modeled using SolidWorks 2020 (Dassault Systèmes) and contained a set of demountable column holders and a replaceable monolithic packing. The upper and lower column holders included a screw thread pair with a hollow cylindrical chamber for filling the monolithic packing, which was stacked as four interlacing cuboids [4.5 mm (length) × 0.5 mm (width) × 0.4 mm (height), with an interstitial distance of 0.5 mm] in each layer and arranged layer by layer with a 60° twisting angle (Figure 1B). Fittings for a standard 10–32 flat-bottom male connector were designed as loading and outlet ports. The cone-shaped ends of the extraction chamber and monolithic packing were designed to function as a flow distributor and collector. Figure S1 illustrates the detailed dimensions of the designed SPE column. A DLP 3D printer (MiiCraft 50) was used to fabricate the column holders and monolithic packing using commercial resins (BV-007A; MiiCraft) and the prepared stimuli-responsive polymer resins, respectively, with a curing time of 4.5 s for a 100 μm *z*-axis resolution (total fabrication time: 102 min; total weight: 35.1 g; material cost: US$16.7). The assembled device was fitted with two flat-bottom male connectors (P-840 and P-844, IDEX Health & Science) with a short PTFE tubing (0.02 in. i.d.; Figure 1C) and washed with 0.5% HNO_3_ for at least 8 h to remove uncured resin components and contaminant metal ions prior to the experiments (Figure 1D).

### Methods and Apparatus

Metal-ion extraction and sample-matrix removal were performed using the SPE column with the 4D-printed dual-responsive monolithic packing in four steps (Table S1). First, the sample was conditioned to the optimal acidity (10 mM phosphate buffer; pH 8.0) and loaded into the SPE column using a peristaltic pump (Miniplus 3, Gilson; loading flow rate: 0.5 mL min^–1^; sample volume: 0.5 mL; Figure S2A). Second, the residual sample matrices in the SPE column were evacuated using an air stream (evacuation flow rate: 0.5 mL min^–1^; evacuation volume: 0.5 mL; Figure S2B). Third, the metal ions extracted by the SPE column were eluted using 0.5% (v/v) HNO_3_ [thermostated in a perfluoroalkoxyalkane bottle using a digital chilling/heating dry bath (EchoTherm, Torrey Pines Scientific); elution flow rate: 1.0 mL min^–1^; elution volume: 1.0 mL] and transported into an ICP-MS system [Agilent 7700×, Agilent Technologies; equipped with Pt sampling and skimmer cones and a Micromist nebulizer (AR35–1-FM04EX, Glass Expansion) fitted to a Scott-type quartz double-pass spray chamber; Figure S2C]. Time-resolved analysis (integration time: 50 ms) and external calibration were performed based on the elution peak areas (integrated using ICP-MS Chromatographic Software C.01.00, Agilent Technologies) at *m*/*z* 55 (Mn), 59 (Co), Ni (60), Zn (64), Cu (65), Cd (114), and Pb (208). Finally, the residual eluent in the SPE column was removed by an air stream (evacuation flow rate: 1.0 mL min^–1^; evacuation volume: 0.5 mL) for the next sample loading without any reconditioning steps (Figure S2D). We automated the sample-pretreatment procedure by integrating the fabricated SPE column, a peristaltic pump, three eight-port valves [C22Z-3188, Valco; programmed through a serial valve interface (SIV-110, Valco)], and the ICP-MS system. The maximal elution peak height (*H*_max_) and elution peak fwhm of the elution profiles of the metal ions were analyzed using OriginPro software (2019b, OriginLab; using Quick Peaks Gadget with a visually corrected baseline) by inputting the raw time-resolved ICP-MS data. *H*_max_/fwhm ratios were used to illustrate the elution peak profiles of the metal ions. To measure the elution-induced [H^+^]- and temperature-responsive changes in the interstitial volume among these interlacing cuboids and confirm the swelling of the monolithic packing, 10 mM phosphate buffer with a pH of 2.0 (protonated polyNIPAM when pH < 3.0;^56−60^ close to p*K*_a1_ of phosphoric acid) and 8.0 (unprotonated polyNIPAM; close to p*K*_a2_ of phosphoric acid) and the temperature lower (10 °C) and higher (40 °C) than the LCST of polyNIPAM was used.

### Sample Collection and Preparation

The reliability of our analytical method was validated by determining the metal ions in one standard reference material [SRM (National Institute of Standards and Technology): 1643f (fresh water)] and three certified reference materials [CRMs: SLRS-5 (untreated river water; collected at the City of Ottawa’s Britannia Water Purification Plant; National Research Council of Canada); CASS-4 (nearshore seawater; collected from Halifax Harbour; National Research Council of Canada); Seronorm Trace Elements Urine L-2 (human urine, reconstituted with 5 mL of deionized water; SERO)]. Analyses of the metal ions in seawater, groundwater, river water, and human urine samples, which were collected after *in situ* filtration using syringe filters (0.45 μm; CHROMAFIL Xtra H-PTFE, Macherey-Nagel) and acidification (0.5% HNO_3_, v/v), as well as spike analyses of the collected samples (spiked concentrations: 0.05 μg L^–1^ for Co, Cd, and Pb; 0.5 μg L^–1^ for Mn, Ni, Cu, and Zn; based on their measured concentrations in these collected samples) were performed to illustrate the analytical applicability of this method. The reference materials and collected samples were neutralized to the optimal sample acidity (pH 8.0) using basified 10 mM phosphate buffer (*e.g*., pH 12.5 for SRM 1643f; pH 11.6 for acidified seawater samples) and analyzed without any other treatment. Statistical comparisons were performed using Student’s two-tailed unpaired *t*-test.

## Results and Discussion

### 4D-Printed Elution-Peak-Guided Dual-Responsive Monolithic Packing

Although an SPE column with a monolith packing of cuboids with small feature sizes and interstitial spaces in a fixed packing volume provides a large surface area that could extract more metal ions, based on previous reports and our experience,^38,46,47,62−64^ vat photopolymerization 3DP usually cannot fabricate objects with enclosed channels (side length) or holes (diameter) smaller than 0.5 mm because of the inherent resolution of 3D printers and the effect of light scattering. Large void volumes among the interlacing cuboids of a monolithic packing could lead to the significant dispersion (dilution) of the eluted metal ions.^42^ Therefore, we used 4DP technology with NIPAM-incorporated photocurable resins to fabricate an SPE column with a [H^+^]/temperature dual-responsive monolithic packing (Figure 1) and develop a completely new strategy to reduce the MDLs of metal ions when coupled with ICP-MS determination. In this strategy, the elution-induced [H^+^]- and temperature-responsive changes in the interstitial volumes of the interlacing cuboids of the monolithic packing are used to optimize the elution peak profiles of the studied metal ions.

When metal ions are eluted from monolithic packings for ICP-MS determination, their total elution peak areas are merely based on the extraction efficiency of the packing; however, their elution peak profiles could vary owing to differences in their transport through the interstitial volume of the monolithic packing and changes in their desorption kinetics, which could contribute to diverse *H*_max_ and fwhm values. Reports suggested that peak height and/or peak fwhm dominated the peak performance,^23,24,65^ but a large amount of tabular peak height and fwhm data might not be easily understood. Therefore, we combined these two characteristics and used their ratio (*H*_max_/fwhm) to simplify the representation of the interactive effects of peak height and fwhm on the elution peak profiles of the metal ions, especially for the changes in both the elution peak areas (extraction efficiencies) and elution peak profiles (*H*_max_ and fwhm) of metal ions when adjusting geometric parameters of the monolithic packing. Under the same elution peak area, a higher *H*_max_/fwhm value indicates a sharper and narrower elution peak. When the extraction efficiencies of the packing for metal ions differ, an increase in *H*_max_/fwhm suggests that the increase in *H*_max_ is greater than that in the elution peak fwhm,^23,24^ indicating the potential for improving the signal-to-noise ratio and lowering MDLs. To demonstrate that the elution peak profiles of the investigated metal ions can be optimized by using the 4D-printed dual-responsive monolithic packing, we selected the experimental conditions based on the *H*_max_/fwhm values of the metal ions rather than their elution peak areas.

**Figure 2:**
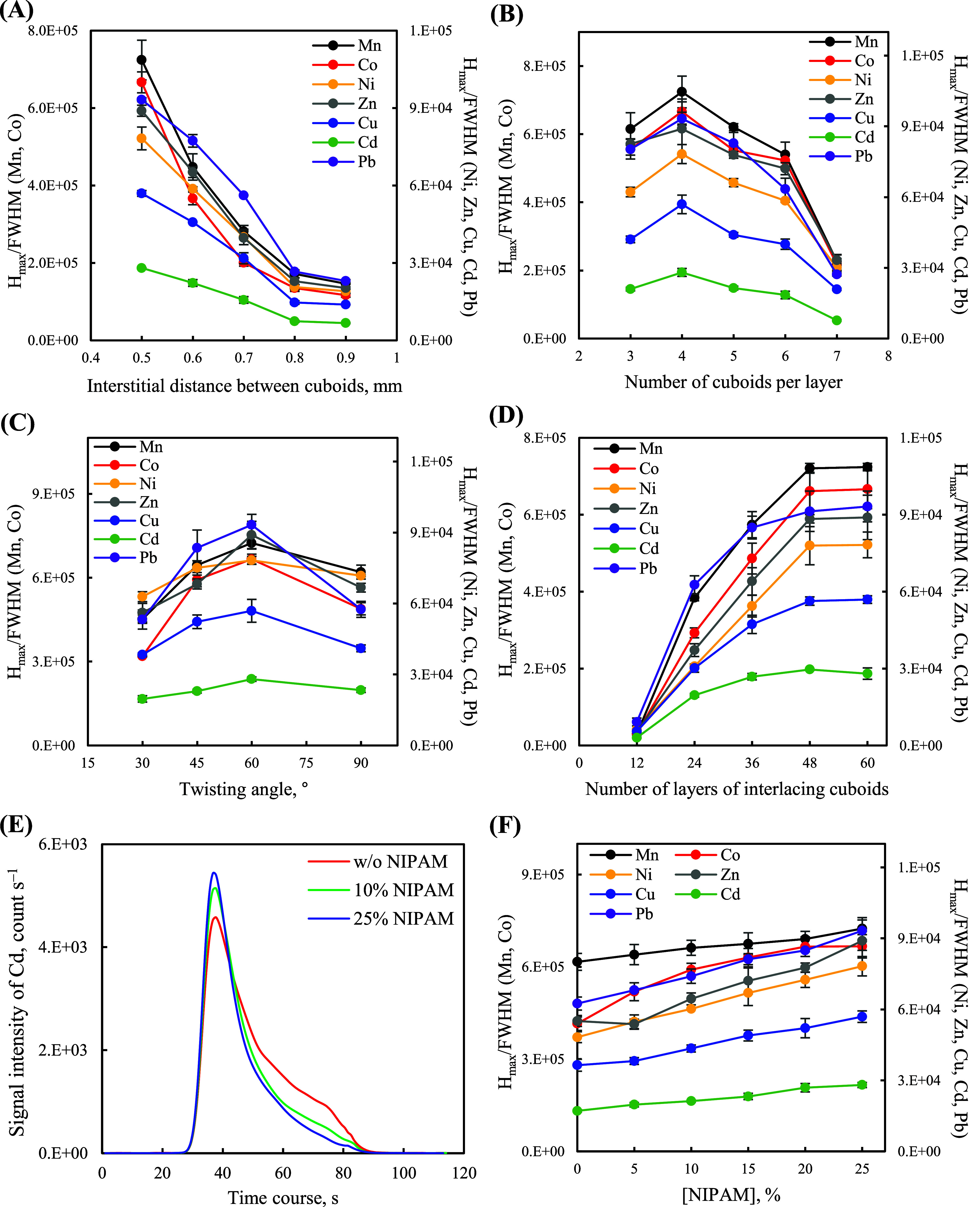
*H*_max_/fwhm values of the investigated metal ions (10 μg L^–1^) plotted with respect to the (A) interstitial distance between cuboids, (B) the number of cuboids per layer, (C) twisting angle, (D) the number of layers of interlacing cuboids, and (F) concentration of NIPAM incorporated in the photocurable resins (eluent: 0.5% HNO_3_ solution). (E) Elution profiles of Cd ions (10 μg L^–1^) plotted with respect to the concentration of NIPAM incorporated in the photocurable resins (eluent: 0.5% HNO_3_ solution). The error bars represent standard deviations (*n* = 6).

Figure 2A indicates that the *H*_max_/fwhm values of the metal ions but not their peak areas (Figure S3A) increased significantly when the interstitial distance between interlacing cuboids was decreased from 0.9 to 0.5 mm because larger interstitial volumes lead to the significant dispersion of the metal ions when eluted under the same flow rate. The increasing *I*_max_/fwhm values of the metal ions potentially revealed the sharper (higher peak height) and narrower (lower peak fwhm) elution peaks under the same elution peak area (signal intensity). Hence, we fixed the interstitial distance between interlacing cuboids at 0.5 mm to optimize the design of the monolithic packing. Figure 2B reveals that the *H*_max_/fwhm values of the metal ions increased when the number of cuboids per layer was increased from 3 to 4 mainly because of the increase in the elution peak areas (Figure S3B) but decreased thereafter because of the trade-off between the increase in the elution peak areas of the metal ions and their dispersion (larger fwhms are induced by increasing the interstitial volume among interlacing cuboids). Figures 2C and S3C indicate that the twisting angle of the interlacing cuboids is optimal at 60°, possibly because of lower shear forces,^47,66^ as indicated by the *H*_max_/fwhm values and elution peak areas of the metal ions. Figure 2D shows that the *H*_max_/fwhm values of the metal ions leveled off when the number of layers of interlacing cuboids was greater than 48 because of the trade-off between the increase in the elution peak areas of the metal ions (Figure S3D) and their dispersion (*H*_max_ and fwhm increased proportionally). In summary, both the signal intensities (elution peak areas) of the metal ions and the interstitial volumes among interlacing cuboids (dispersion of the eluted metal ions) affected the elution peak profiles of the metal ions. Therefore, we fixed the packing dimension at four cuboids per layer, the twisting angle at 60°, and the number of interlacing cuboid layers at 48 as optimal conditions for fabricating the elution-peak-guided monolithic packing.

Because the fabrication of a monolithic packing stacked as interlacing cuboids with an interstitial distance smaller than 0.5 mm is often difficult, we incorporated NIPAM into the photocurable resins to fabricate the stimuli-responsive monolithic packing and enable stimuli-responsive reductions in the interstitial volume among cuboids to improve the elution peak profiles of the metal ions. Interestingly, when we increased the concentration of the incorporated NIPAM in the photocurable resins, the elution peak profiles of the metal ions became sharper and narrower (Figure 2E) under the same elution peak area (Figures S3E), and their *H*_max_/fwhm values increased significantly when they were eluted with 0.5% HNO_3_ solution (Figure 2F). By contrast, the *H*_max_/fwhm values leveled off when elution was performed with EDTA solution [10 mg L^–1^ (with 0.1% ammonium hydroxide); Figure S3F]. Although these two eluents were able to effectively elute the extracted metal ions from the monolithic packing (Figures S3E and S3G), elution using HNO_3_ solution led to electrostatic repulsion among the protonated units (NH_2_^+^; when pH < 3.0)^56−60^ of polyNIPAM and induced the swelling of the interlacing cuboids, resulting in smaller interstitial volumes among cuboids [from 0.45 ± 0.02 (pH 8.0, 40 °C) to 0.35 ± 0.02 mL (pH 2.0, 40 °C); Figure S3H] and improvements in the elution peak profiles of the metal ions. Owing to limitations in the solubility of NIPAM in the prepared photocurable resins and their printability, we fixed the concentration of the incorporated NIPAM at 25% as an optimal condition for the 4DP of the elution-peak-guided stimuli-responsive monolithic packing. In addition, we selected 0.5% HNO_3_ solution, based on the invariant signal intensities (Figure S4A) and the optimal *H*_max_/fwhm values (Figure S4B) of the metal ions, as the eluent to improve the elution peak profiles of the metal ions. These results testified that the *I*_max_/fwhm values were suitable to display the interactive effects of peak height and fwhm on the elution peak profiles for evaluating the parameters that contributed to both invariant and variant signal intensities of the metal ions.

### Extraction of Metal Ions Using the SPE Column with the 4D-Printed Dual-Responsive Monolithic Packing

After optimizing the design and fabrication of the [H^+^]/temperature dual-responsive monolithic packing, we evaluated the operating conditions to optimize the elution peak profiles of the metal ions and/or maximize the sensitivity of our analytical method. Figure S4C shows their pH-dependent extraction profiles, but Figure S4D reveals the pH-independent profiles of the *H*_max_/fwhm values of the metal ions (both *H*_max_ and fwhm increased proportionally, except at pH 2.0). The signal intensities of the metal ions increased when the sample pH was increased from 3.0 to 8.0, presumably because the interactions between the positively charged metal-ion species (Mn^2+^, Co^2+^, Ni^2+^, Cu^2+^, CuOH^+^, Zn^2+^, ZnOH^+^, Cd^2+^, CdCl^+^, Pb^2+^, PbOH^+^, based on modeling using Visual MINTEQ 3.1^67,68^) and partially negatively charged C=O groups and N atoms of polyNIPAM (Figure S5) were minimally influenced by hydronium ions. However, the signal intensities leveled off when the samples were eluted under basic conditions, presumably because the interactions between uncharged [MnCl_2_, Co(OH)_2_, Ni(OH)_2_, Cu(OH)_2_, CuCl_2_, Zn(OH)_2_, ZnCl_2_, Cd(OH)_2_, CdCl_2_, Pb(OH)_2_, PbCl_2_] and negatively charged [Co(OH)_3_^–^, Ni(OH)_3_^–^, Cu(OH)_3_^–^, Zn(OH)_3_^–^, Cd(OH)_3_^–^, Pb(OH)_3_^–^] metal-ion species were unaltered by the partially negatively charged C=O groups and N atoms of polyNIPAM. The stimuli-responsive material was utilized for the first time to fabricate a monolithic packing for metal-ion extraction. Based on the signal intensities and elution peak profiles of the metal ions, we selected a pH of 8.0 as the optimal sample acidity. Through loading of a sample containing the metal ions (10 mg L^–1^) into the SPE column with the 4D-printed dual-responsive monolithic packing to, respectively, acquire the sample volume that provided the saturated signal intensities of the extracted metal ions, we measured the adsorption capacities (corrected by the extraction efficiencies) of the packing for these ions to be 51.7 μg Mn, 52.0 μg Co, 53.4 μg Ni, 58.8 μg Zn, 62.7 μg Cu, 54.3 μg Cd, and 58.6 μg Pb cm^–2^. These values correspond to the amounts of metal ions that could be extracted from a 1.0 mL sample at concentrations ranging from 414 (Mn) to 502 (Cu) mg L^–1^ [surface area of the monolithic packing (provided by Solidworks 2020): 8.0 cm^2^].

**Figure 3:**
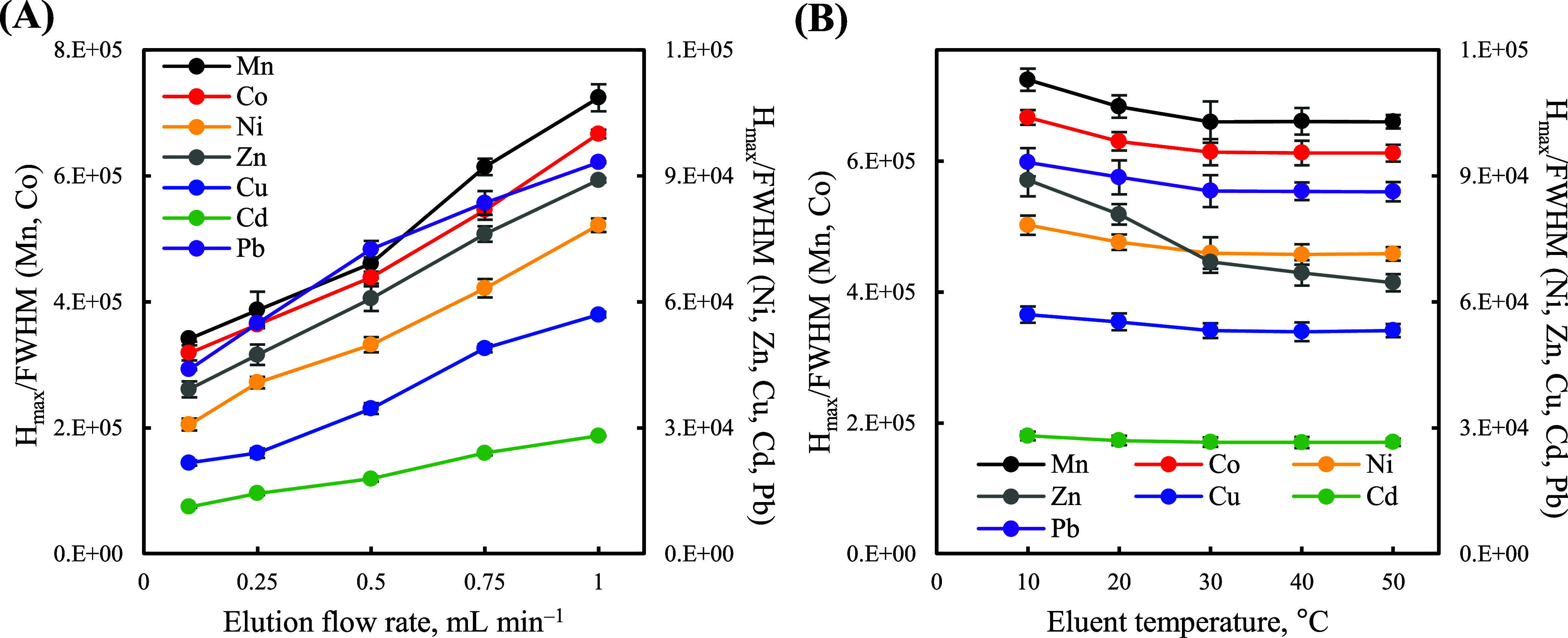
*H*_max_/fwhm values of the investigated metal ions (10 μg L^–1^) plotted with respect to the (A) elution flow rate and (B) eluent temperature. The error bars represent standard deviations (*n* = 6).

Because of the submillimeter-sized interstitial distance between interlacing cuboids, the sample loading and elution flow rates of the SPE column easily reached 1.0 mL min^–1^ without significant flow resistance (back-pressure: < 1.0 psi).^38,44,46^Figure S4E shows that the signal intensities of the metal ions slightly declined when the sample loading flow rate was increased (>90% of the signal intensities of the metal ions were maintained at a loading flow rate of 0.75 mL min^–1^ compared with those at a loading flow rate of 0.1 mL min^–1^). Thus, we selected 0.5 mL min^–1^ as the optimal sample loading flow rate. When the elution flow rate was increased, the signal intensities of the metal ions, as expected, markedly declined (Figure S4F) owing to reductions in their detection time; however, the *H*_max_/fwhm values of the metal ions increased significantly (Figure 3A), which is important in reducing their dispersion when the interstitial distance between interlacing cuboids is fixed. In addition, the NIPAM-incorporated interlacing cuboids swelled when eluted with an eluent with a temperature below the LCST of polyNIPAM (approximately 27 °C after incorporation into the tBA/HDDA resins;^54,55,69,70^Figure S6), resulting in smaller interstitial volumes among cuboids [from 0.45 ± 0.02 mL (pH 8.0, 40 °C) to 0.39 ± 0.02 mL (pH 8.0, 10 °C); Figure S3H] and improvements in the elution peak profiles of the metal ions (Figure 3B) but not their elution peak areas (Figure S4G). Owing to the [H^+^]/temperature dual-responsive properties of the 4D-printed monolithic packing, the interstitial distance between interlacing cuboids clearly decreased (Figure S7), with the total interstitial volume (Figure S3H) decreasing from 0.45 ± 0.02 mL (pH 8.0, 40 °C) to 0.30 ± 0.02 mL (pH 2.0, 10 °C), thereby substantially sharpening the elution peaks of the metal ions. Based on the elution peak profiles of the metal ions, we selected 0.5% HNO_3_ solution as the eluent (Figures S4A and S4B), 10 °C as the eluent temperature, and 1.0 mL min^–1^ as the elution flow rate to elute the extracted metal ions from the dual-responsive monolithic packing. Under optimal elution conditions, the signal intensities of the tested metal ions (10 μg L^–1^) declined to less than 5% of the maximum response of their respective elution profiles within 44 s (Figure S8A) without significant carry-over effects (Figure S8B). We also adopted an elution volume of 1.0 mL to elute the metal ions completely.

Next, we evaluated the consistency of the SPE column with the 4D-printed dual-responsive monolithic packing. The relative standard deviations (RSDs) of the signal intensities of the metal ions, which were measured from seven columns (interdevice variations), were all less than 6.7%, which suggests that the fabricated SPE columns were highly consistent in extracting the metal ions. We also evaluated the durability of the SPE column. The fluctuations (RSDs) of the daily calibration slopes for the metal ions (interday variations) were all less than 15.7% when the same column was used for up to 57 days (to construct the calibration curves and perform the analyses of the metal ions in the reference materials and spike analyses of real samples; Figure S8C), thus confirming the applicability of the SPE column for long-term operation. Figures S4H and S4I reveal that both common ions [K^+^ (1000 mg L^–1^), Ca^2+^ (1000 mg L^–1^), Mg^2+^ (1000 mg L^–1^), Fe^3+^ (100 mg L^–1^), Al^3+^ (100 mg L^–1^), HCO_3_^–^ (500 mg L^–1^), SO_4_^2–^ (2000 mg L^–1^), and Br^–^ (500 mg L^–1^); spiked recoveries: 95–107%] and dissolved salts (>85% of the signal intensities of the metal ions were maintained in samples with salinities of up to 4.5% NaCl) had negligible effects on the extraction of the metal ions, revealing good tolerance when exploiting electrostatic polyNIPAM–metal-ion interactions for metal-ion extraction. These findings collectively indicate that the SPE column with the 4D-printed elution-peak-guided dual-responsive monolithic packing is suitable for the interference-free determination of the investigated metal ions in real samples with high salt contents.

### Analytical Characteristics

**Figure 4:**
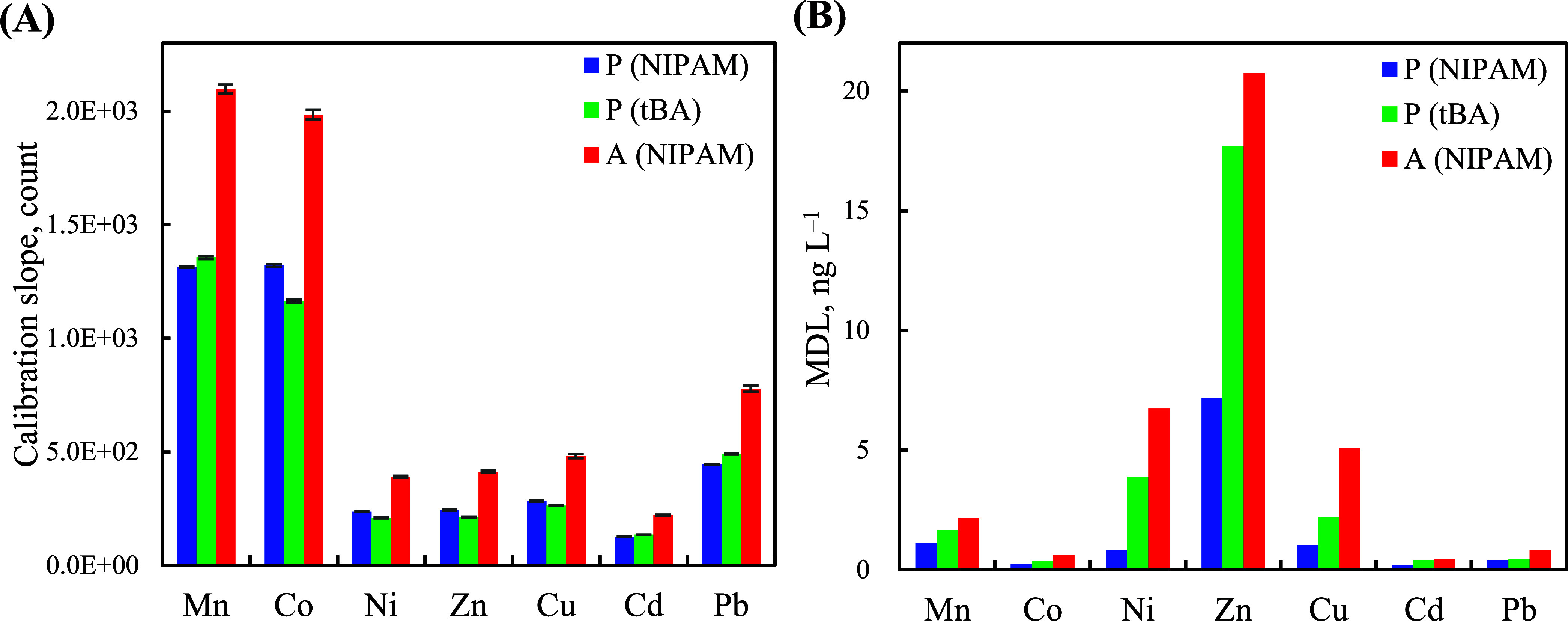
Effect of the optimization mode on the (A) calibration slopes and (B) MDLs of the investigated metal ions. The error bars in (A) represent the standard error of the slope. The MDLs in panel (B) were calculated as 3 times the standard deviation of the baseline noise from seven blank measurements for each metal ion.

After optimizing the operation of the SPE column with the 4D-printed dual-responsive monolithic packing, we systematically studied the effects of optimizing the elution peak profiles of the metal ions on MDL reduction using ICP-MS analysis. Using the optimization results, we constructed calibration curves of the metal ions under three experimental conditions (Tables S1 and S2), namely, peak-profile mode with incorporating NIPAM [P (NIPAM); the number of interlacing cuboid layers: 48; elution flow rate: 1.0 mL min^–1^; eluent temperature: 10 °C; Figure S9A,B], peak-profile mode without incorporating NIPAM [P (tBA); same conditions applied in P (NIPAM) mode; Figure S9C,D], and peak-area mode with incorporating NIPAM [A (NIPAM); the number of interlacing cuboid layers: 60; elution flow rate: 0.25 mL min^–1^; eluent temperature: 40 °C; Figure S9E,F]. Interestingly, although A (NIPAM) mode provided the highest calibration slopes (based on elution peak areas; Figure 4A) of the metal ions, P (NIPAM) mode provided the lowest MDLs (0.2–7.2 ng L^–1^; based on 3 times the standard deviation of the baseline noise from seven blank measurements) when compared with those of P (tBA) (MDLs: 0.4–17.7 ng L^–1^) and A (NIPAM) (MDLs: 0.5–20.7 ng L^–1^) modes (Figure 4B). These results suggest that the 4D-printed stimuli-responsive monolithic packing improves the elution peak profiles of the metal ions and enhances their signal-to-noise ratios but not their elution peak areas, which is beneficial for achieving lower MDLs, especially for metal ions with measurable blank levels (*e.g*., Mn, Ni, Zn, and Cu).

Under the optimized operating conditions [P (NIPAM) mode] for the SPE column with the 4D-printed dual-responsive monolithic packing (Table S2; sample volume: 0.5 mL; sample throughput: 17.1 h^–1^), the absolute extraction efficiencies of the metal ions ranged from 91.9 to 95.1% [ratios of the elution peak areas (10 μg L^–1^) with and without extraction; Table 1], their correlation coefficients (*R*) in the working range were all greater than 0.9997 (Figure S9A,B), and the enhancement factors (EFs) ranged from 13.0 to 19.3 [ratios of *H*_max_ (10 μg L^–1^) after and before extraction^71,72^]. Compared with previously reported 3D-printed SPE devices^38−48^ and commercial SPE devices^73−75^ for sample pretreatment and trace-element determination (Table S3), the SPE column with the 4D-printed dual-responsive monolithic packing developed in this study provided superior analytical characteristics, including much lower MDLs (under the minimum sample volume), higher throughput, and greater extraction capacities. To validate the applicability of our analytical method for the reliable determination of metal ions in natural water and urine samples, the concentrations of the investigated metal ions in an SRM and CRMs were measured. Tables S4 and S5 reveal that the measured concentrations of the metal ions agreed with their certified values [relative errors: from −5.3 to +3.6%; all *p* values: > 0.1817 (significant difference: *p* < 0.05)]. Moreover, the spike recoveries of the metal ions in natural water samples and a urine sample were in the ranges of 96–103, and 98–101%, respectively. These results confirm the tolerance of the SPE column with the dual-responsive monolithic packing toward real sample matrices and the applicability of our analytical method for the reliable and sensitive determination of the investigated metal ions in real natural water and urine samples.

**Table 1:** Analytical Characteristics of the Developed Analytical Method using the SPE Column with the 4D-Printed Dual-Responsive Monolithic Packing

element	working range, ng L^–1^	calibration curve	*R*	MDL, ng L^–1^	extraction efficiency, %	EF^b^
^55^ Mn	50–5000	*y* = 1313[Mn]^a^ + 38583	1.0000	1.1	93.2	19.3
^59^Co	1–100	*y* = 1320[Co]^a^ + 12514	0.9999	0.2	94.3	19.3
^60^Ni	50–5000	*y* = 237[Ni]^a^ + 7324	0.9999	0.8	92.4	14.6
^64^Zn	50–5000	*y* = 243[Zn]^a^ + 98916	0.9999	7.2	91.9	13.0
^65^Cu	50–5000	*y* = 283[Cu]^a^ + 11852	0.9997	1.0	92.8	13.0
^114^Cd	1–100	*y* = 127[Cd]^a^ + 769	0.9999	0.2	95.1	18.2
^208^Pb	1–100	*y* = 446[Pb]^a^ + 1275	0.9999	0.4	94.9	16.9

a ng L^–1^.

b Ratio of the elution peak heights after and before extraction (10 μg L^–1^).

## Conclusions

In this study, 4DP technologies with stimuli-responsive materials were utilized for the first time to fabricate an SPE column featuring a [H^+^]/temperature dual-responsive monolithic packing to optimize the elution peak profiles and reduce the MDLs of the proposed analytical method for the reliable and sensitive determination of Mn, Co, Ni, Cu, Zn, Cd, and Pb ions in complex real samples. Compared with previously reported SPE schemes with enhanced extraction efficiencies for target analytes, our device and analytical method present several attractive characteristics. (i) The designed dual-responsive monolithic packing was directly 4D-printed and packed in a 3D-printed SPE column without labor-intensive and time-consuming fabrication procedures, thus showing the great capability of 4DP in fabricating analytical devices with desirable stimuli-responsive properties. (ii) The SPE scheme and analytical method proposed in this study were optimized by improving the elution peak profiles of the metal ions but not their elution peak areas. Our findings confirmed the feasibility of optimizing elution peak profiles to reduce MDLs for metal-ion analysis. (iii) The stimuli-responsive behaviors of the fabricated monolithic packing could be readily adjusted by varying the elution conditions to optimize the elution peak profiles of the target metal ions or method sensitivity. This characteristic provides an alternative to conventional SPE sorbents that have been modified to optimize their extraction efficiencies. Although studies introducing 4DP and stimuli-responsive materials to fabricate analytical devices with optimized geometric features and stimuli-responsive shape programming are rare, we believe that 4DP technologies will continue to unlock new possibilities for advancing the functionality and performance of conventional analytical devices to meet future analytical requirements.

## Supporting Information Available

Detailed dimensions of the designed SPE column and monolithic packing; schematic representation of the automatic analytical system; relative signal intensities of these extracted metal ions plotted with respect to the interstitial distance between cuboids, the number of cuboids per layer, twisting angle, the number of layers of interlacing cuboids, concentration of NIPAM incorporated in the photocurable resins, concentration of HNO_3_ in the eluent, sample acidity, sample loading flow rate, elution flow rate, eluent temperature, concentration of NaCl, and interference ions; *H*_max_/fwhm values of the investigated metal ions plotted with respect to the concentration of NIPAM incorporated in the photocurable resins, concentration of HNO_3_ in the eluent, and sample acidity; effects of the eluent pH and temperature on the interstitial volume among these interlacing cuboids in the monolithic packing; infrared spectrum and thermal analysis of the cured NIPAM-incorporated photocurable resins; photographs of the 4D-printed dual-responsive monolithic packing under the elution conditions of 10 mM PB (pH 8.0, 40 °C) and 0.5% HNO_3_ solution (10 °C); elution profiles and temporal responses of these metal ions in the analytical system; daily calibration slopes of these metal ions for the same fabricated SPE column used for up to 57 days; calibration plots of these metal ions; operating sequence and conditions of the automatic analytical system; analytical characteristics of reported 3D-printed SPE devices for sample pretreatment and facilitating trace-element determination; characteristics of reference materials measured using the analytical method; analytical data of real samples measured using the analytical method (PDF).
