# Supplementary material for: 4D-Printed Elution-Peak-Guided Dual-Responsive Monolithic Packing for the Solid-Phase Extraction of Metal Ions
Source: Anal Chem. 2024 Feb 21;96(11):4469–78. doi: 10.1021/acs.analchem.3c04961 (PMC10955517; doi:10.1021/acs.analchem.3c04961)
Supplement: Supplementary file 1 — ac3c04961_si_001.pdf [file ac3c04961_si_001.pdf]

Supporting Information

4D-Printed Elution-Peak-Guided Dual-Responsive Monolithic Packing for the Solid-Phase Extraction of Metal Ions

Wen-Hsiu Tsai and Cheng-Kuan Su*

Department of Chemistry, National Chung Hsing University, Taichung City 402, Taiwan, R.O.C.

Corresponding Author

*E-mail: cksu@nchu.edu.tw

Table of Content

Page S-5	**Figure S1.** Detailed dimensions of the designed SPE column. The grey parts were fabricated using BV-007A photocurable resins; the blue part was fabricated using NIPAM-incorporated photocurable resins. Units: mm.
Page S-6	**Figure S2.** Schematic representation of the automatic analytical system using the SPE column with the 4D-printed dual-responsive monolithic packing. (A) The conditioned sample (pH 8.0) was loaded (0.5 mL min^{-1}) into the SPE column for extraction of the metal ions. (B) Residual sample matrices were evacuated (0.5 mL min^{-1}) using an air stream. (C) The extracted metal ions were eluted (1.0 mL min^{-1}) with a 0.5% HNO_3 solution and delivered into the ICP-MS system for time-resolved analysis. (D) The residual eluent in the SPE column was replaced by an air stream (1.0 mL min^{-1}) for loading of the next sample. V1, V2, and V3: two-position, eight-port electric switching valves; unmarked arrow: outflow of liquid waste.
Page S-8	**Figure S3.** Relative signal intensities of these extracted metal ions (10 μg L^{-1}) plotted with respect to the (A) interstitial distance between cuboids, (B) number of cuboids per layer, (C) twisting angle, (D) number of layers of interlacing cuboids, (E) concentration of NIPAM incorporated in the photocurable resins (eluent: 0.5% HNO_3 solution), and (G) concentration of NIPAM incorporated in the photocurable resins (eluent: 0.1% ammonium hydroxide solution containing 10 mg L^{-1} EDTA). (F) H_{max}/FWHM values of the investigated metal ions (10 μg L^{-1}) plotted with respect to the concentration of NIPAM incorporated in the photocurable resins (eluent: 0.1% ammonium hydroxide solution containing 10 mg L^{-1} EDTA). (H) Effects of the eluent pH and temperature on

	the interstitial volume among these interlacing cuboids in the fabricated monolithic packing. Data in (A)–(E) and (G) have been normalized to the respective maxima of these metal ions for each parameter.
Page S-10	**Figure S4.** Relative signal intensities of these extracted metal ions (10 μg L^{-1}) plotted with respect to the (A) concentration of HNO_3 in the eluent, (C) sample acidity, (E) sample loading flow rate, (F) elution flow rate, (G) eluent temperature, (H) concentration of NaCl, and (I) interference ions. H_{max}/FWHM values of the investigated metal ions (10 μg L^{-1}) plotted with respect to the (B) concentration of HNO_3 in the eluent and (D) sample acidity. Data in (A), (C), and (E)–(I) have been normalized to the respective maxima of these metal ions for each parameter.
Page S-12	**Figure S5.** Infrared spectrum of the cured NIPAM-incorporated photocurable resins, recorded using a Fourier transform infrared spectrometer (Tensor 27, Bruker).
Page S-13	**Figure S6.** Thermal analysis (differential scanning calorimetry) of the cured NIPAM-incorporated photocurable resins, recorded using a thermal analyzer (HT-2, Mettler Toledo).
Page S-14	**Figure S7.** Photographs of the 4D-printed dual-responsive monolithic packing under the elution conditions of (A) 10 mM PB (pH 8.0, 40 °C) and (B) 0.5% HNO_3 solution (10 °C), analyzed using ImageJ software.
Page S-16	**Figure S8.** (A) Elution profiles and (B) temporal responses of these metal ions (10 μg L^{-1}) in the analytical system employing the SPE column with the 4D-printed dual-responsive monolithic packing. (C) Daily calibration slopes of these metal ions for the same fabricated SPE column used for up to 57 days.

Page S-17	**Figure S9.** (A), (C), (E) Calibration plots of Mn, Ni, Zn, and Cu (50–5000 ng L^{-1}), and (B), (D), (F) calibration plots of Co, Cd, and Pb (1–100 ng L^{-1}). (A) and (B): peak-profile mode with incorporating NIPAM [P (NIPAM)]; (C) and (D): peak-profile mode without incorporating NIPAM [P (tBA)]; (E) and (F): peak-area mode with incorporating NIPAM [A (NIPAM)].
Page S-19	**Table S1.** Operating sequence of the automatic analytical system employing the SPE column with the 4D-printed dual-responsive monolithic packing
Page S-20	**Table S2.** Optimized conditions for the automatic analytical system employing the SPE column with the 4D-printed dual-responsive monolithic packing
Page S-22	**Table S3.** Analytical characteristics of reported 3D-printed SPE devices and commercial SPE devices for sample pretreatment and facilitating trace-element determination
Page S-24	**Table S4.** Characteristics of reference materials measured using the analytical method employing the SPE column with the 4D-printed dual-responsive monolithic packing (n = 5)
Page S-26	**Table S5.** Analytical data of real samples measured using the analytical method with the SPE column with the 4D-printed dual-responsive monolithic packing (n = 5)

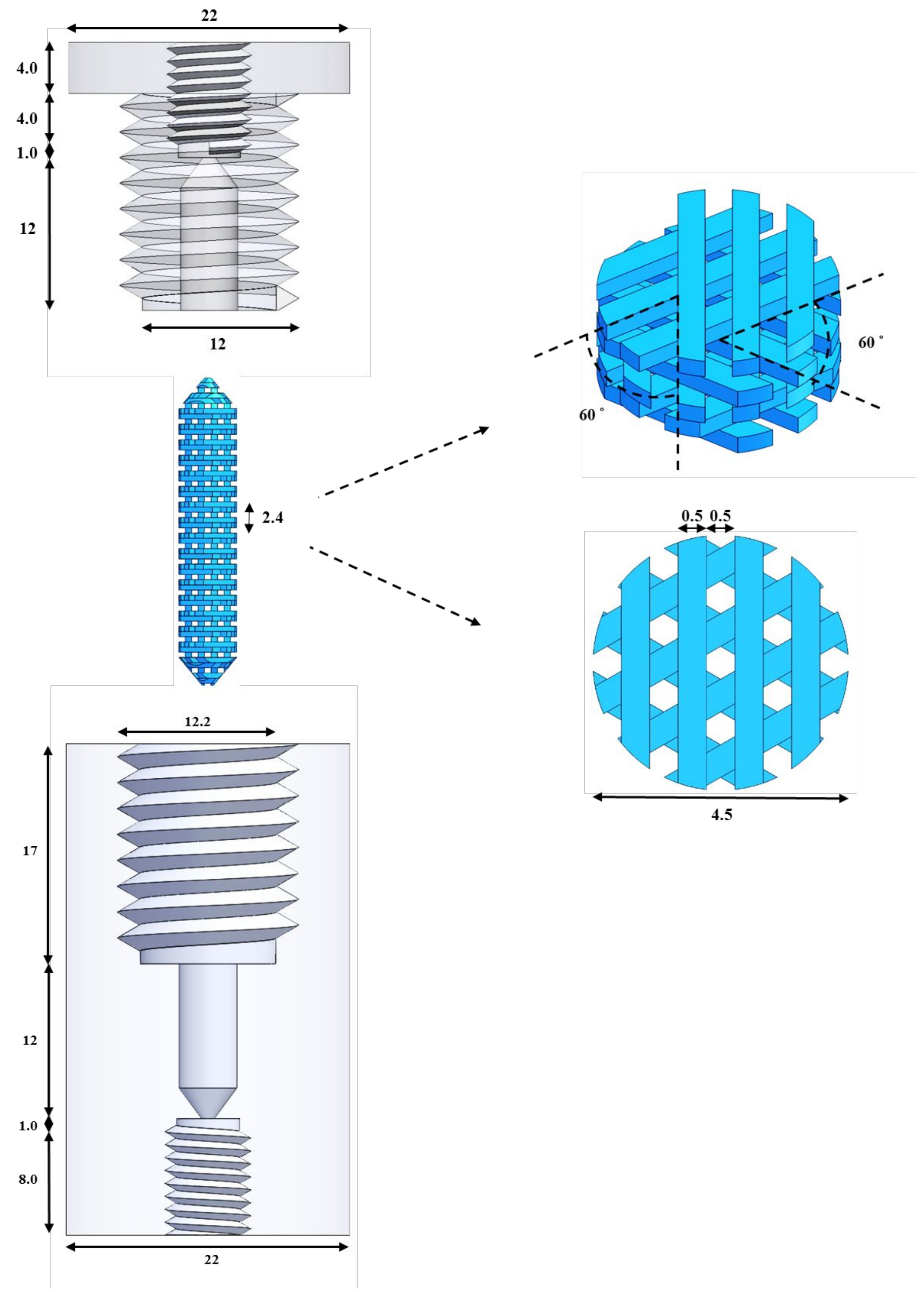

Figure S1. Detailed dimensions of the designed SPE column. The grey parts were fabricated using BV-007A photocurable resins; the blue part was fabricated using NIPAM-incorporated photocurable resins. Units: mm.

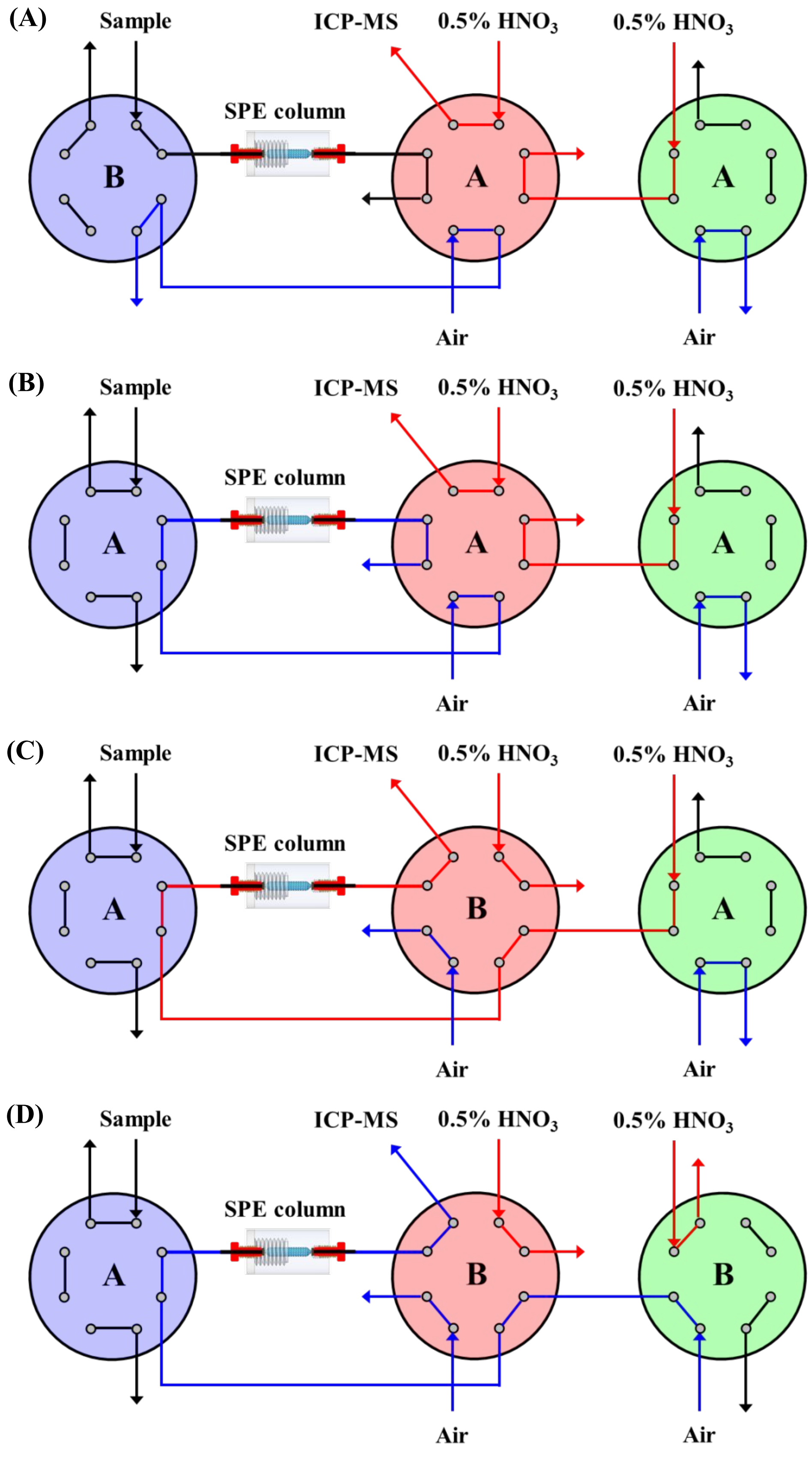
(A)
Sample
ICP-MS
0.5% HNO3
0.5% HNO3
SPE column
B
A
A
Air
Air
(B)
Sample
ICP-MS
0.5% HNO3
0.5% HNO3
SPE column
A
A
A
Air
Air
(C)
Sample
ICP-MS
0.5% HNO3
0.5% HNO3
SPE column
A
B
A
Air
Air
(D)
Sample
ICP-MS
0.5% HNO3
0.5% HNO3
SPE column
A
B
B
Air
Air

Figure S2. Schematic representation of the automatic analytical system using the SPE column with the 4D-printed dual-responsive monolithic packing. (A) The conditioned sample (pH 8.0) was loaded (0.5 mL min^{-1}) into the SPE column for extraction of the metal ions. (B) Residual sample matrices were evacuated (0.5 mL min^{-1}) using an air stream. (C) The extracted metal ions were eluted (1.0 mL min^{-1}) with a 0.5% HNO_3 solution and delivered into the ICP-MS system for time-resolved analysis. (D) The residual eluent in the SPE column was replaced by an air stream (1.0 mL min^{-1}) for loading of the next sample. V1, V2, and V3: two-position, eight-port electric switching valves; unmarked arrow: outflow of liquid waste.

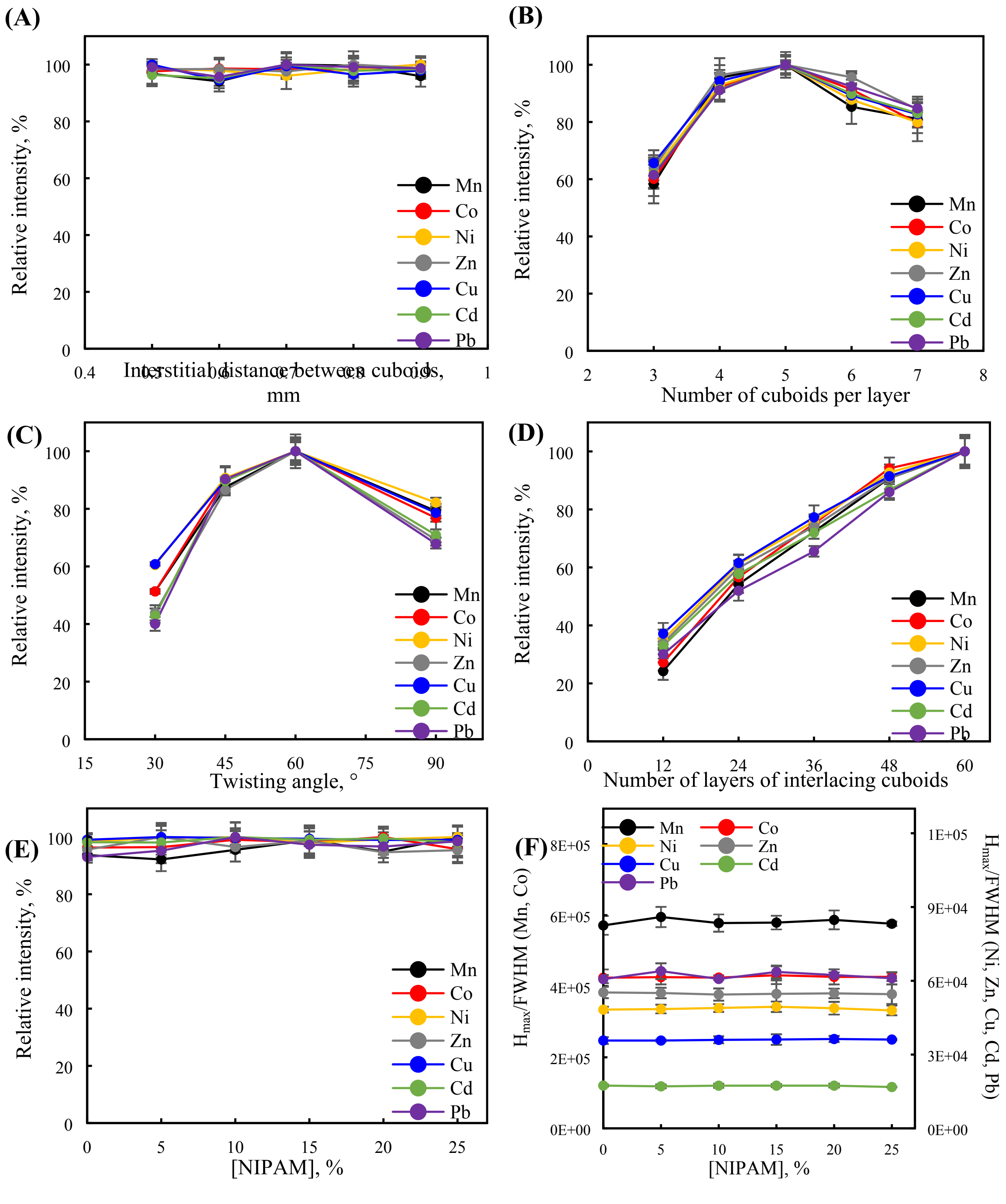
(A)
Relative intensity, %
Interstitial distance between cuboids, mm
Mn
Co
Ni
Zn
Cu
Cd
Pb
(B)
Relative intensity, %
Number of cuboids per layer
(C)
Relative intensity, %
Twisting angle, °
(D)
Relative intensity, %
Number of layers of interlacing cuboids
(E)
Relative intensity, %
[NIPAM], %
(F)
H_{max}/FWHM (Mn, Co)
H_{max}/FWHM (Ni, Zn, Cu, Cd, Pb)
[NIPAM], %

(G)

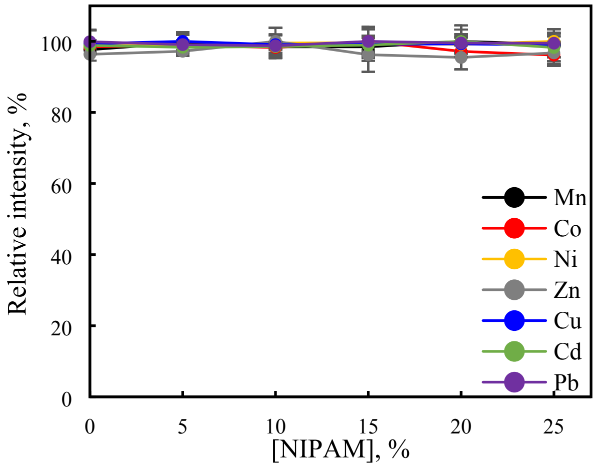

(H)

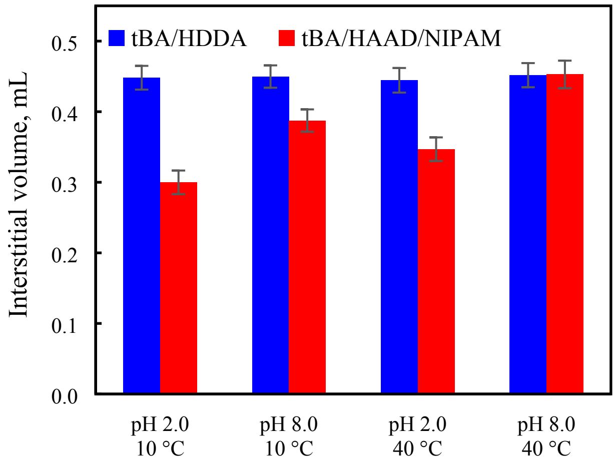

Figure S3. Relative signal intensities of these extracted metal ions (10 μg L^{-1}) plotted with respect to the (A) interstitial distance between cuboids, (B) number of cuboids per layer, (C) twisting angle, (D) number of layers of interlacing cuboids, (E) concentration of NIPAM incorporated in the photocurable resins (eluent: 0.5% HNO_3 solution), and (G) concentration of NIPAM incorporated in the photocurable resins (eluent: 0.1% ammonium hydroxide solution containing 10 mg L^{-1} EDTA). (F) H_{max}/FWHM values of the investigated metal ions (10 μg L^{-1}) plotted with respect to the concentration of NIPAM incorporated in the photocurable resins (eluent: 0.1% ammonium hydroxide solution containing 10 mg L^{-1} EDTA). (H) Effects of the eluent pH and temperature on the interstitial volume among these interlacing cuboids in the fabricated monolithic packing. Data in (A)–(E) and (G) have been normalized to the respective maxima of these metal ions for each parameter. The error bars represent standard deviations (*n* = 6).

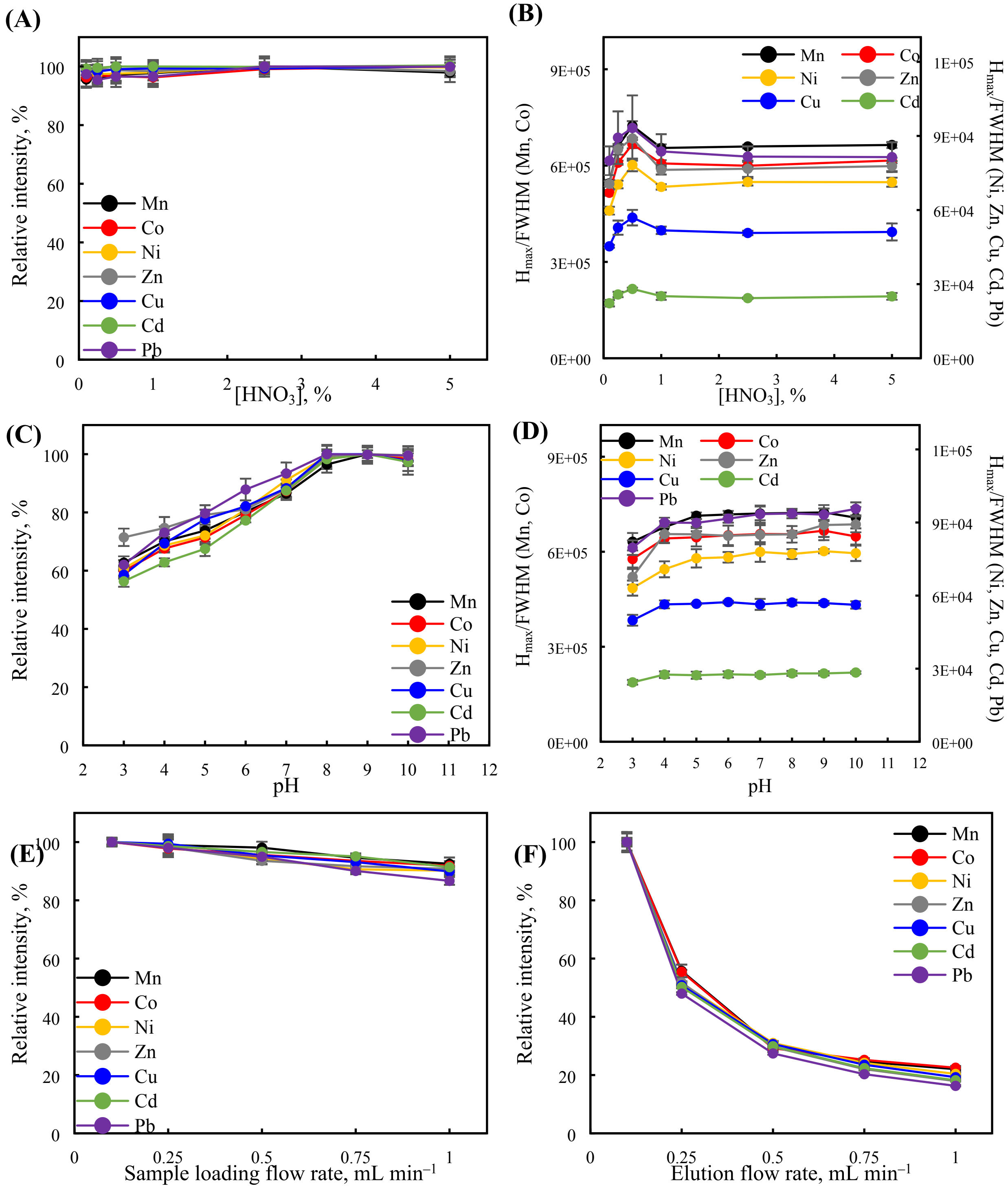
(A)
Relative intensity, %
[HNO3], %
(B)
Hmax/FWHM (Mn, Co)
Hmax/FWHM (Ni, Zn, Cu, Cd, Pb)
[HNO3], %
(C)
Relative intensity, %
pH
(D)
Hmax/FWHM (Mn, Co)
Hmax/FWHM (Ni, Zn, Cu, Cd, Pb)
pH
(E)
Relative intensity, %
Sample loading flow rate, mL min−1
(F)
Relative intensity, %
Elution flow rate, mL min−1
Mn
Co
Ni
Zn
Cu
Cd
Pb

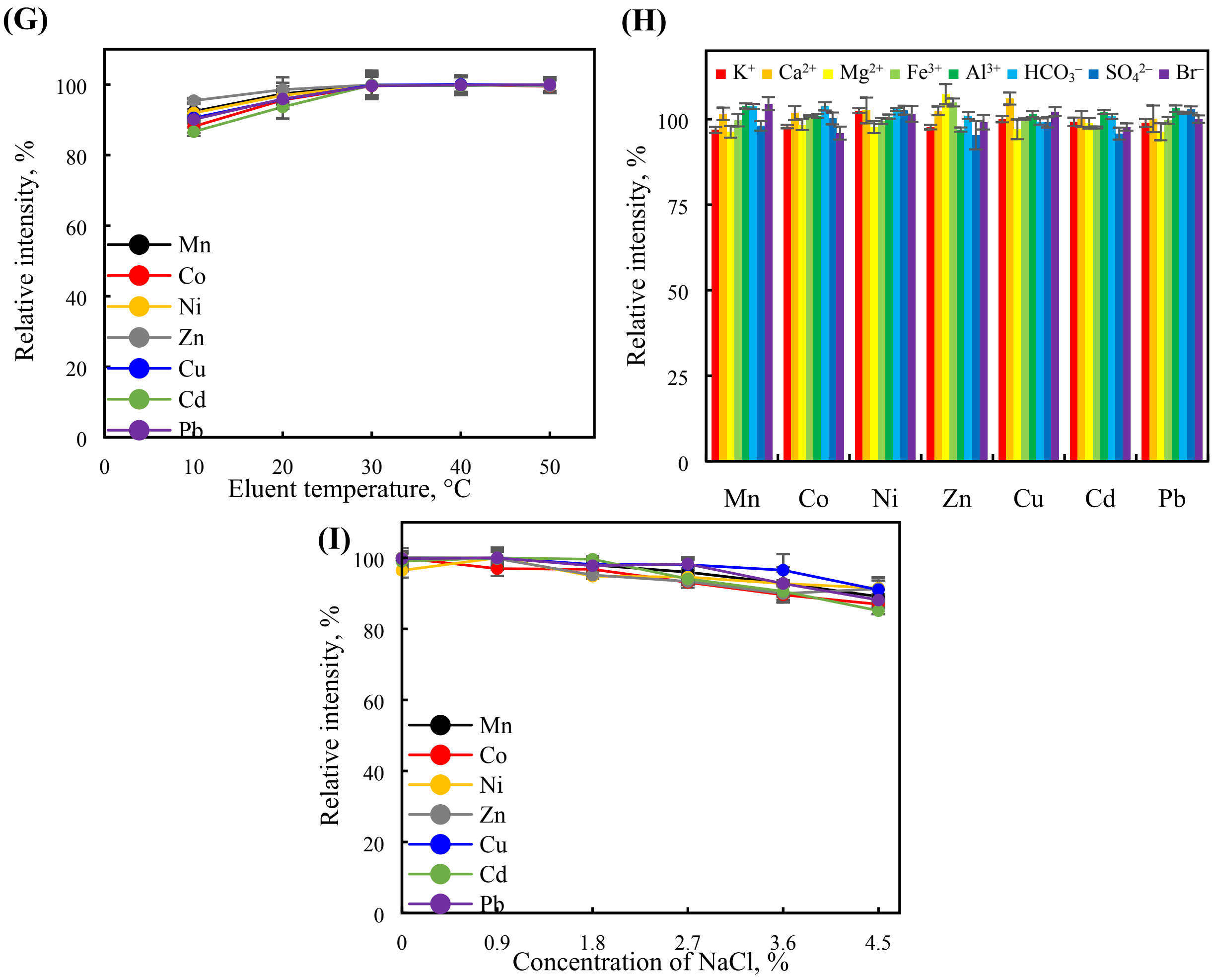

Figure S4. Relative signal intensities of these extracted metal ions (10 μg L^{-1}) plotted with respect to the (A) concentration of HNO_3 in the eluent, (C) sample acidity, (E) sample loading flow rate, (F) elution flow rate, (G) eluent temperature, (H) concentration of NaCl, and (I) interference ions. H_{max}/FWHM values of the investigated metal ions (10 μg L^{-1}) plotted with respect to the (B) concentration of HNO_3 in the eluent and (D) sample acidity. Data in (A), (C), and (E)–(I) have been normalized to the respective maxima of these metal ions for each parameter. The error bars represent standard deviations (n = 6).

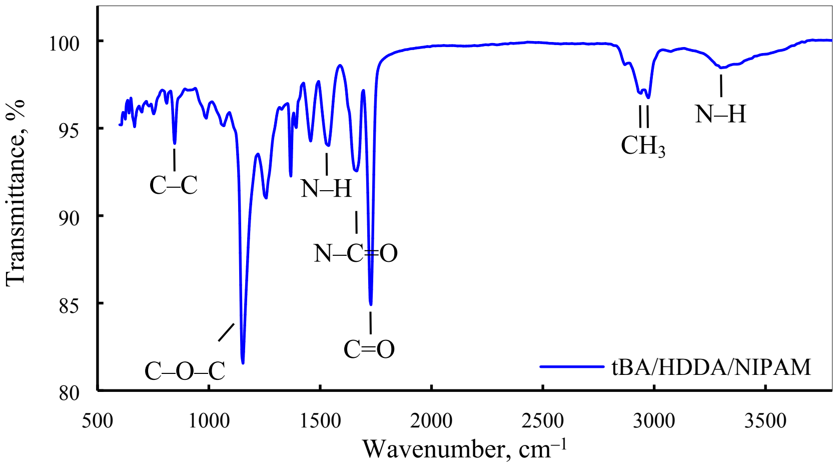

Figure S5. Infrared spectrum of the cured NIPAM-incorporated photocurable resins, recorded using a Fourier transform infrared spectrometer (Tensor 27, Bruker).

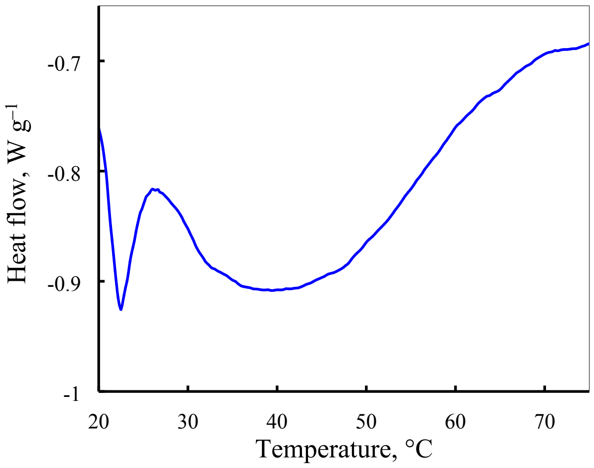

Figure S6. Thermal analysis (differential scanning calorimetry) of the cured NIPAM-incorporated photocurable resins, recorded using a thermal analyzer (HT-2, Mettler Toledo).

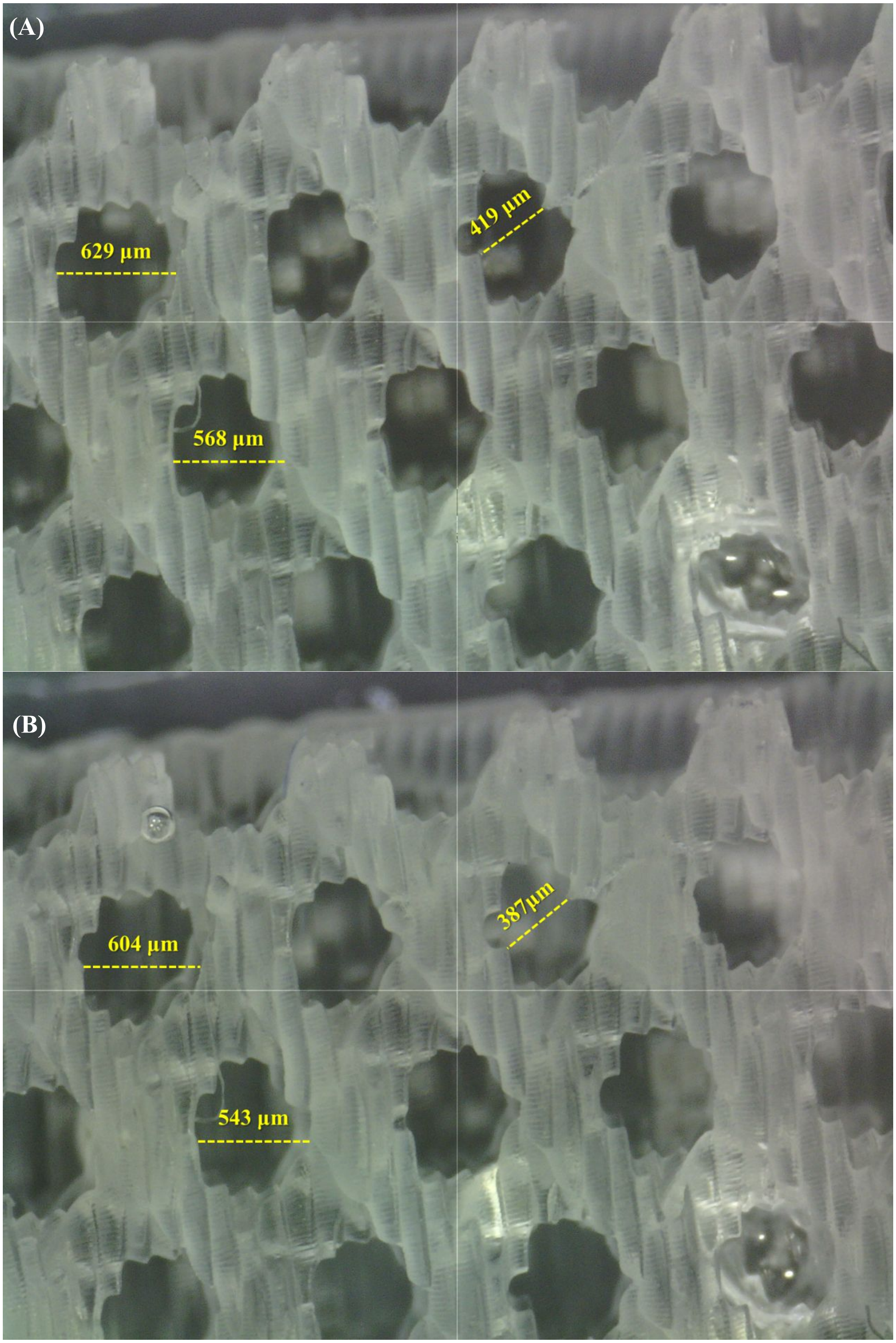

(A)
629 µm
419 µm
568 µm
(B)
604 µm
387µm
543 µm

Figure S7. Photographs of the 4D-printed dual-responsive monolithic packing under the elution conditions of (A) 10 mM PB (pH 8.0, 40 °C) and (B) 0.5% HNO_3 solution (10 °C), analyzed using ImageJ software. Treatment time: 1 min.

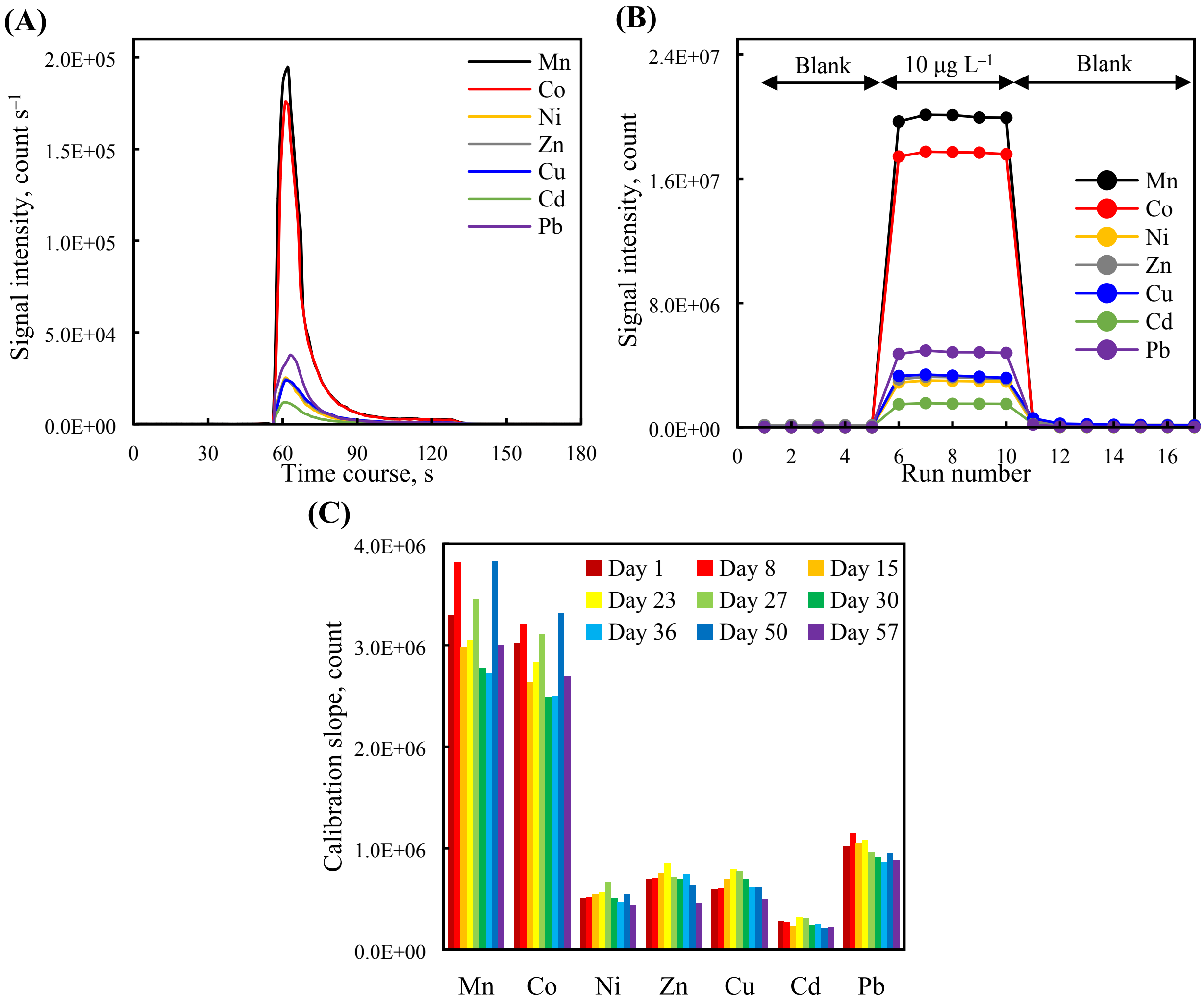

Figure S8. (A) Elution profiles and (B) temporal responses of these metal ions (10 μg L^{-1}) in the analytical system employing the SPE column with the 4D-printed dual-responsive monolithic packing. (C) Daily calibration slopes of these metal ions for the same fabricated SPE column used for up to 57 days. The fluctuations (RSDs) of the calibration slopes for these metal ions were 12.8% for Mn, 10.8% for Co, 12.0% for Ni, 15.7% for Zn, 14.2% for Cu, 13.9% for Cd, and 9.7% for Pb.

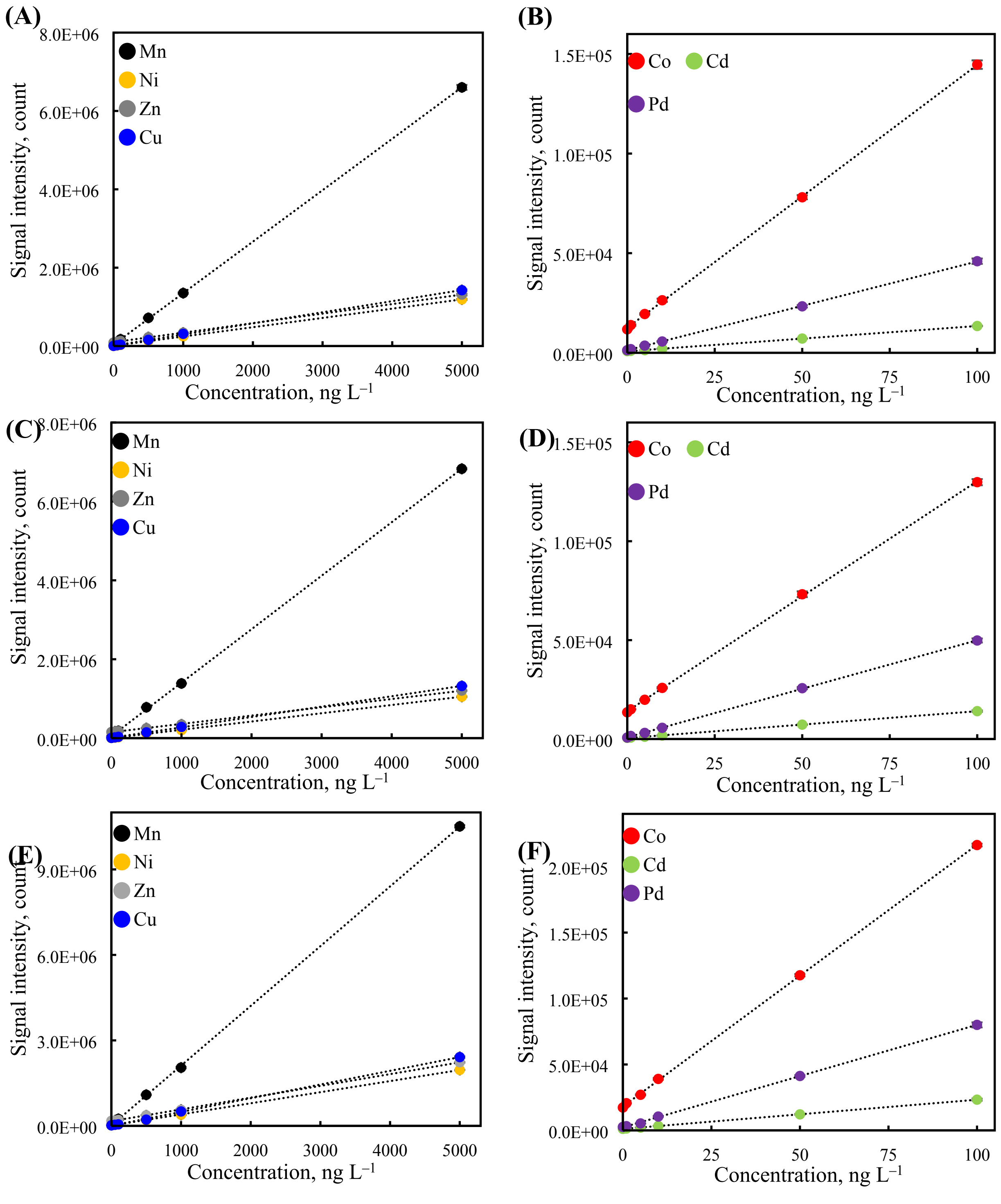

Figure S9. (A), (C), (E) Calibration plots of Mn, Ni, Zn, and Cu (50–5000 ng L^{-1}), and (B), (D), (F) calibration plots of Co, Cd, and Pb (1–100 ng L^{-1}). The error bars represent standard deviations (n = 6). (A) and (B): peak-profile mode with incorporating NIPAM [P (NIPAM)]; (C) and (D):

peak-profile mode without incorporating NIPAM [P (tBA)]; (E) and (F): peak-area mode with incorporating NIPAM [A (NIPAM)].

Table S1. Operating sequence of the automatic analytical system employing the SPE column with the 4D-printed dual-responsive monolithic packing

Step	Valve position (1 → 3)	Time interval for P (NIPAM)	Time interval for P (tBA)	Time interval for A (NIPAM)	Function
1 (Figure S2A)	BAA	0:00–1:00	0:00–1:00	0:00–1:00	loading of the conditioned sample into the SPE column
2 (Figure S2B)	AAA	1:00–2:00	1:00–2:00	1:00–2:00	evacuating the SPE column with an air stream to remove sample matrices
3 (Figure S2C)	ABA	2:00–3:00	2:00–3:00	2:00–6:00	eluting these extracted metal ions and delivering them into the ICP-MS system
4 (Figure S2D)	ABB	3:00–3:30	3:00–3:30	6:00–8:00	replacing the residual eluent with an air stream for next loading

Table S2. Optimized conditions for the automatic analytical system employing the SPE column with the 4D-printed dual-responsive monolithic packing

**Design and fabrication of the dual-responsive monolithic packing**			
	P (NIPAM)	P (tBA)	A (NIPAM)
Dimension of cuboids (L × W × H), mm	0.5 × 4.5 × 0.4	0.5 × 4.5 × 0.4	0.5 × 5.5 × 0.4
Interstitial distance, mm	0.5	0.5	0.5
Number of cuboids per layer	4	4	5
Twisting angle, °	60	60	60
Number of layers of interlacing cuboids	48	48	60
Concentration of NIPAM, %	25	0	25

**Sample pretreatment scheme when employing the SPE column with the 4D-printed dual-responsive monolithic packing**			
	P (NIPAM)	P (tBA)	A (NIPAM)
Sample condition	pH 8.0, 40 °C	pH 8.0, 40 °C	pH 8.0, 40 °C
Conditioning buffer	10 mM PB	10 mM PB	10 mM PB
Sample loading flow rate, mL min^{-1}	0.5	0.5	0.5
Sample volume, mL	0.5	0.5	0.5
Evacuation medium for sample	Air	Air	Air
Evacuation flow rate, mL min^{-1}	0.5	0.5	0.5
Evacuation volume, mL	0.5	0.5	0.5
Eluent	0.5% HNO_3	0.5% HNO_3	0.5% HNO_3
Eluent temperature, °C	10	10	40
Elution flow rate, mL min^{-1}	1.0	1.0	0.25
Elution volume, mL	1.0	1.0	1.0
Evacuation medium for eluent	Air	Air	Air

Evacuation flow rate, mL min^{-1}	1.0	1.0	0.25
Evacuation volume, mL	0.5	0.5	0.5

**ICP-MS system**	
ICP mass spectrometer	Agilent 7700x
Plasma forward power	1500 W
Ar gas flow rate	
Plasma gas	15 L min^{-1}
Auxiliary gas	0.9 L min^{-1}
Carrier gas	0.85 L min^{-1}
Makeup gas	0.19 L min^{-1}
Sampling cone	Pt, 1-mm orifice
Skimmer cone	Pt, 0.4-mm orifice
Analysis mode	Time-resolved analysis
Integration time	50 ms
Isotopes monitored	^{55}Mn, ^{59}Co, ^{60}Ni, ^{64}Zn, ^{65}Cu, ^{114}Cd, ^{208}Pb

Table S3. Analytical characteristics of reported 3D-printed SPE devices and commercial SPE devices for sample pretreatment and facilitating trace-element determination

Device	Analytes	Sample volume, mL	Throughput, h^{-1}	MDL, ng L^{-1}	Extraction efficiency, %	Capacity, µg cm^{-2}	Reference
SPE column with 4D-printed dual-responsive monolithic packing	Mn, Co, Ni, Cu, Zn, Cd, Pb	0.5	17.1	0.2–7.2	91.9–95.1	51.7–62.7	this study
minicolumn packed with Nobias PA1 resins	Mn, Fe, Co, Ni, Cu, Zn, Cd, Pb	40	0.7	0.01–4.99[a]	--	--	73
seaFAST chelation column (Nobias PA1 resins)	Mn, Fe, Co, Ni, Cu, Zn	9.0	6.9	0.02–1.05[a]	--	--	74
3D-printed right-angled-turned knotted reactor	Mn, Co, Ni, Cu, Zn, Cd, Pb	4.0	15.8	0.1–5.6	73.9–85.9	–	47
3D-printed column with porous PA6 monolithic packing	Mn, Co, Ni, Cu, Zn, Cd, Pb	1.0	11.5	0.2–7.7	94.3–98.5	27.8–66.4	45
3D-printed column with porous monolithic packing	Mn, Co, Ni, Cu, Zn, Cd, Pb	1.0	12	0.3–6.7	99.2–99.8	5.3–14.3	44
3D-printed preconcentrator	Mn, Ni, Cu, Zn, Cd, Pb	1.0	10	0.3–18.0	–	8.9 (Cu)	38

4D-printed temperature-controlled flow-actuated SPE device	Mn, Co, Ni, Cu, Zn, Cd, Pb	5.0	25	0.7–22.1	77.5–96.0	0.85 (Cd)	46
3D-printed SPE column incorporating TiO_2 NP–coated porous monolith	Cr(III), Cr(VI), As(III), As(V), Se(IV), Se(VI)	2.0	15	0.7–32.3	84.5–98.3	45.4–75.9	48
3D-printed minicolumn	Fe(II), Fe(III)	1.0	7.5	1–2	65–87	0.43–2.53	42
3D-printed TiO_2 NP–incorporated minicolumn	As(III), As(V), Se(IV), Se(VI)	0.5	10	4–128	14–62	0.40–0.76	40
Chelex-100 column	V, Mn, Co, Ni, Cu, Zn, As, Cd, Pb	2.0	5.5	5–345	19–100	--	75
3D-printed filter scavenger	Hg(II)	495	0.6	37	95.2	222.4[b]	43
3D-printed disk-based SPE device	Cr(VI)	16	6	62.5	–	–	39
3D-printed SPE device	U(VI)	9.0	3	500	93	–	41

[a]: analyzed with magnetic sector inductively coupled plasma mass spectrometry detection

[b]: $mg\ g^{-1}$

Table S4. Characteristics of reference materials measured using the analytical method employing the SPE column with the 4D-printed dual-responsive monolithic packing (n = 5)

		CASS-4	SLRS-5	1643f	Trace Elements Urine L-2
Mn	Certified value, µg L^{-1}	2.78 ± 0.19	0.081 ± 0.006	37.14 ± 0.60	10.9 ± 2.2
	Measured value, µg L^{-1}	2.68 ± 0.09	0.08 ± 0.001	37.19 ± 0.06	10.53 ± 0.27
	Relative error, %	–3.5	+1.9	+0.1	–3.4
	p value	0.3186	0.3557	0.8575	0.7186
Co	Certified value, µg L^{-1}	0.026 ± 0.003	0.05*a*	25.30 ± 0.17	10.6 ± 2.1
	Measured value, µg L^{-1}	0.03 ± 0.001	0.05 ± 0.001	25.24 ± 0.15	10.69 ± 0.07
	Relative error, %	+3.6	0	–0.2	+0.9
	p value	0.4996	--	0.5703	0.9261
Ni	Certified value, µg L^{-1}	0.314 ± 0.030	0.476 ± 0.064	59.8 ± 1.4	41.3 ± 8.3
	Measured value, µg L^{-1}	0.32 ± 0.01	0.47 ± 0.003	59.49 ± 0.69	41.16 ± 0.72
	Relative error, %	+1.8	–0.8	–0.5	–0.3
	p value	0.6826	0.8390	0.6687	0.9709
Zn	Certified value, µg L^{-1}	0.381 ± 0.057	0.845 ± 0.095	74.4 ± 1.7	1338 ± 269
	Measured value, µg L^{-1}	0.39 ± 0.01	0.84 ± 0.01	73.48 ± 1.10	1374.18 ± 10.17
	Relative error, %	+2.8	–0.7	–1.2	+2.7
	p value	0.7370	0.8925	0.3394	0.7714
Cu	Certified value, µg L^{-1}	0.592 ± 0.055	17.4 ± 1.3	21.66 ± 0.71	22*a*
	Measured value, µg L^{-1}	0.57 ± 0.02	17.54 ± 0.38	21.46 ± 0.21	22.37 ± 0.23
	Relative error, %	–4.2	+0.8	–0.9	+1.7
	p value	0.4250	0.8230	0.5626	--
Cd	Certified value, µg L^{-1}	0.026 ± 0.003	0.0060 ± 0.0014	5.89 ± 0.13	4.9 ± 0.2
	Measured value, µg L^{-1}	0.02 ± 0.002	0.01 ± 0.0003	6.03 ± 0.17	4.81 ± 0.18

	Relative error, %	–5.3	–2.5	+2.4	–1.8
	p value	0.5524	1.0000	0.1817	0.4759
Pb	Certified value, µg L^{-1}	0.0098 ± 0.0036	0.081 ± 0.006	18.488 ± 0.084	90.7 ± 18.3
	Measured value, µg L^{-1}	0.01 ± 0.0004	0.08 ± 0.002	18.40 ± 0.31	86.64 ± 1.00
	Relative error, %	–3.4	–2.8	–0.5	–4.5
	p value	0.8577	0.4996	0.5571	0.6337

[a]: Information value

Table S5. Analytical data of real samples measured using the analytical method with the SPE column with the 4D-printed dual-responsive monolithic packing (n = 5)

		Seawater	River water	Ground water	Urine
Mn	Measured conc., µg L^{-1}	0.21 ± 0.002	0.22 ± 0.01	0.17 ± 0.01	0.12 ± 0.004
	Conc. after spiking, µg L^{-1}	0.73 ± 0.03	0.73 ± 0.01	0.66 ± 0.01	0.62 ± 0.03
	Spike recovery,[a] %	104	102	98	100
Co	Measured conc., µg L^{-1}	0.03 ± 0.001	0.13 ± 0.004	0.15 ± 0.001	0.03 ± 0.001
	Conc. after spiking, µg L^{-1}	0.08 ± 0.003	0.18 ± 0.01	0.20 ± 0.002	0.08 ± 0.003
	Spike recovery,[b] %	103	99	97	99
Ni	Measured conc., µg L^{-1}	0.56 ± 0.02	3.20 ± 0.14	0.55 ± 0.01	0.57 ± 0.02
	Conc. after spiking, µg L^{-1}	1.06 ± 0.03	3.70 ± 0.07	1.06 ± 0.02	1.07 ± 0.02
	Spike recovery,[a] %	100	99	101	100
Zn	Measured conc., µg L^{-1}	1.26 ± 0.05	0.94 ± 0.03	1.37 ± 0.02	13.32 ± 0.15
	Conc. after spiking, µg L^{-1}	1.77 ± 0.06	1.44 ± 0.04	1.88 ± 0.03	13.82 ± 0.03
	Spike recovery,[a] %	102	99	101	101
Cu	Measured conc., µg L^{-1}	0.17 ± 0.004	0.44 ± 0.01	0.92 ± 0.02	0.49 ± 0.01
	Conc. after spiking, µg L^{-1}	0.65 ± 0.02	0.93 ± 0.02	1.43 ± 0.02	0.98 ± 0.03
	Spike recovery,[a] %	96	97	101	98
Cd	Measured conc., µg L^{-1}	0.01 ± 0.0003	ND	ND	0.01 ± 0.0002
	Conc. after spiking, µg L^{-1}	0.06 ± 0.004	0.05 ± 0.002	0.05 ± 0.001	0.06 ± 0.003
	Spike recovery,[b] %	103	98	98	99
Pb	Measured conc., µg L^{-1}	0.004 ± 0.0002	0.09 ± 0.004	0.04 ± 0.001	ND
	Conc. after spiking, µg L^{-1}	0.05 ± 0.003	0.14 ± 0.01	0.09 ± 0.003	0.05 ± 0.003
	Spike recovery,[b] %	99	100	103	100

[a]: Spiked concentration: 0.5 µg L^{-1}; [b]: spike concentration: 0.05 µg L^{-1}.
